# Synapse integrity and function: Dependence on protein synthesis and identification of potential failure points

**DOI:** 10.3389/fnmol.2022.1038614

**Published:** 2022-12-13

**Authors:** Laurie D. Cohen, Tamar Ziv, Noam E. Ziv

**Affiliations:** ^1^Technion Faculty of Medicine, Rappaport Institute and Network Biology Research Laboratories, Haifa, Israel; ^2^Smoler Proteomics Center, Lokey Interdisciplinary Center for Life Sciences & Engineering, Technion, Haifa, Israel

**Keywords:** synaptic integrity, synaptic tenacity, protein synthesis inhibitors, proteomics, neurodegeneration

## Abstract

Synaptic integrity and function depend on myriad proteins - labile molecules with finite lifetimes that need to be continually replaced with freshly synthesized copies. Here we describe experiments designed to expose synaptic (and neuronal) properties and functions that are particularly sensitive to disruptions in protein supply, identify proteins lost early upon such disruptions, and uncover potential, yet currently underappreciated failure points. We report here that acute suppressions of protein synthesis are followed within hours by reductions in spontaneous network activity levels, impaired oxidative phosphorylation and mitochondrial function, and, importantly, destabilization and loss of both excitatory and inhibitory postsynaptic specializations. Conversely, gross impairments in presynaptic vesicle recycling occur over longer time scales (days), as does overt cell death. Proteomic analysis identified groups of potentially essential ‘early-lost’ proteins including regulators of synapse stability, proteins related to bioenergetics, fatty acid and lipid metabolism, and, unexpectedly, numerous proteins involved in Alzheimer’s disease pathology and amyloid beta processing. Collectively, these findings point to neuronal excitability, energy supply and synaptic stability as early-occurring failure points under conditions of compromised supply of newly synthesized protein copies.

## Introduction

Each and every aspect of synaptic structure and function depends on myriad proteins - labile molecules with finite lifetimes - that need to be continually replaced with freshly synthesized copies. It follows that continuous protein renewal is vital for maintaining the structural integrity and functional properties of synapses. While this need applies to practically all cells and subcellular organelles, synapses, and neurons in general, are uniquely challenged in this respect by the expansive architecture of neurons and the remoteness of most synapses from central biosynthetic centers (e.g., Kleim et al., 2003; Rosenberg et al., 2014; Ziv, 2018; Lottes and Cox, 2020). Specialized solutions to these challenges have evolved in neurons, such as elaborate transport systems and decentralized, distributed protein synthesis and processing systems (e.g., Hafner et al., 2019; Schiapparelli et al., 2019; Biever et al., 2020; for recent reviews see: Maday et al., 2014; Maeder et al., 2014; Glock et al., 2017; Rangaraju et al., 2017; Cioni et al., 2018; Sahoo et al., 2018; Guedes-Dias and Holzbaur, 2019; Holt et al., 2019; Sasaki, 2020; Rizalar et al., 2021; Grochowska et al., 2022; Giandomenico et al., 2022). In addition, the challenge might be partially mitigated by the unusually long lifespans of most neuronal and synaptic proteins (days to weeks) reported in a growing number of studies (Price et al., 2010; Cohen et al., 2013; Hakim et al., 2016; Dörrbaum et al., 2018; Fornasiero et al., 2018; Heo et al., 2018; Mathieson et al., 2018; reviewed in Cohen and Ziv, 2019). Yet, these same studies point to the existence of multiple proteins with much shorter life spans (Sharma et al., 2012; see also Sossin, 2018). It is therefore plausible that certain aspects of synaptic and neuronal physiology are particularly sensitive to the availability of newly synthesized proteins and are thus particularly vulnerable to disruptions in their supply. What might these be? Neuronal excitability? Neurotransmitter recycling? Synaptic persistence? Synaptic tenacity? Which proteins and protein groups are involved?

Here we describe experiments designed to expose neuronal properties and functions that are particularly sensitive to disruptions in the supply of newly synthesized protein copies, and identifying proteins (and groups of proteins) lost early upon such disruptions. To perturb protein replenishment, we used protein synthesis inhibitors (PSIs), fully recognizing that such perturbations are not physiological and doom the neurons to certain death. Nevertheless, our assumption was that careful analyses of failure chronology will expose neuronal functions and processes that most strongly depend on uninterrupted supply of newly synthesized protein copies, and will uncover potential points of failure whose importance for maintaining synaptic and neuronal function in normal as well as pathological circumstances might be currently underappreciated.

## Results

### PSI selection and validation

To expose neuronal properties and functions most sensitive to disruptions in newly synthesized protein supply, we examined how these properties and functions are affected by acute exposure to PSIs. The interpretation of findings based on this approach, however, depends on the efficacy of PSIs in suppressing protein synthesis and the specificity of these effects. We thus set out to measure the efficacy of several commonly used PSIs, and then select the most efficient ones for subsequent experiments. To that end, we used an assay we previously described (Cohen et al., 2020) based on the HaloTag technology and a fusion protein of HaloTag and the fluorescent protein mTurquoise2 (HaloTag-mTurq2). Here, cortical neurons in primary culture expressing this fusion protein were first exposed at saturating concentrations to the non-fluorescent, cell permeable HaloTag ligand CPXH (1-chloro-6-(2-propoxyethoxy) hexane), essentially the HaloTag ligand backbone without a fluorescent group (Cohen et al., 2020). The neurons were then washed and exposed to the fluorescent HaloTag ligand JF635HT (Grimm et al., 2017). Under control conditions, subsequent labeling with JF635HT reflected the binding of JF635HT to HaloTag-mTurq2 synthesized during the elapsed time from CPXH exposure, whereas reduced labeling in the presence of PSIs provides a measure of their protein synthesis inhibition efficacy.

**Figure 1:**
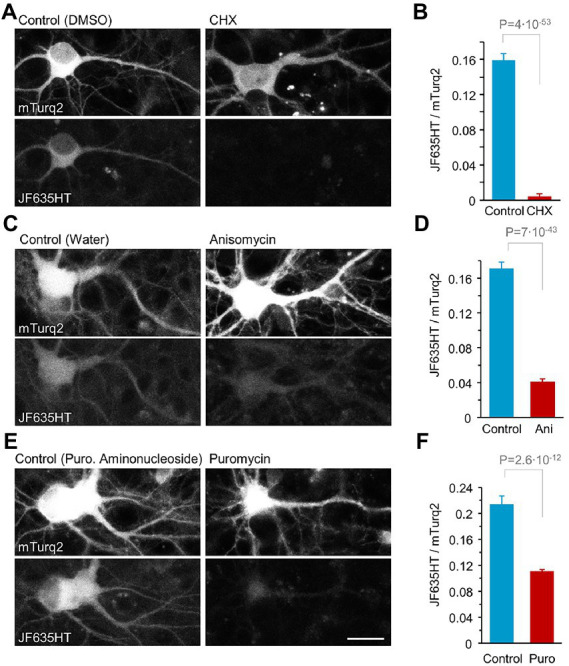
Quantitative assessment of protein synthesis suppression following a 24-hour treatment with three protein synthesis inhibitors used in this study. Cortical neurons expressing HaloTag-mTurq2 were treated with CPXH. Proteins labeled with fluorescent HaloTag ligands following CPXH blocking reflect newly synthesized protein copies. The cells were washed and thereafter exposed to cycloheximide (CHX), anisomycin (ANI), puromycin (PURO) or carrier solution (0.1% DMSO in cell culture media, 0.3% water in cell culture media, or puromycin aminonucleoside, 1 μM final concentration, respectively) for 24 hours followed by JF635HT labeling. **(A)** JF635HT labeling in two such neurons (left: 24-h CHX; right: 24-h carrier solution). **(B)** 24-hour protein synthesis suppression by CHX resulted in a nearly 40-fold (39.2) reduction in JF635HT labeling intensity. Data are from 81 and 178 neurons, CHX and carrier solutions respectively, pooled from 4 replicates (cell culture wells) per condition from 2 separate experiments (separate cell culture preparations from different rat pup litters). **(C,D)** Same as in **(A,B)** but for treatment with ANI. 24-h protein synthesis suppression by ANI resulted in a 4-fold (4.17) reduction in JF635HT labeling intensity. Data are from 155 and 201 neurons, 6 and 7 replicates per condition, ANI and carrier solutions respectively, from 3 separate experiments. **(E,F)** Same as in **(A,B)** but for treatment with PURO. 24-h protein synthesis suppression by PURO resulted in a nearly 2-fold (1.93) reduction in JF635HT labeling intensity. Data are from 169 and 83 neurons, 6 and 4 replicates per condition from 3 and 2 separate experiments, PURO and carrier solutions, respectively. Scale bar, 20 μm. Average + SEM, t-test assuming unequal variances. SEM and t test based on numbers of neurons (analyses by replicates provided in Supplementary Figure S1). Data in A and B taken from Cohen et al. (2020).

Three PSIs, at commonly used concentrations, were tested in this fashion: (1) Cycloheximide (CHX) which blocks protein synthesis by interfering with tRNA translocation during elongation, at a concentration of 100 μg/ml (355 μM), (2) Anisomycin (ANI) which prevents peptide bond formation by inhibiting peptidyl transferase activity, at 25 μM, and (3) Puromycin which acts as an aminoacyl-tRNA analog leading to premature elongation termination, at 1 μM (for references on inhibitors’ mode of action and working concentrations, see: Yarmolinsky and Haba, 1959; Ennis and Lubin, 1964; Nathans, 1964; Wettstein et al., 1964; Grollman, 1967; Zinck et al., 1995; Fonseca et al., 2006; Abbas et al., 2009; Schneider-Poetsch et al., 2010; Villers et al., 2012; Abbas, 2013; Signer et al., 2014; tom Dieck et al., 2015; Gawron et al., 2016; Nixon-Abell et al., 2016; Chang et al., 2017; Li and Götz, 2017; Liu et al., 2017; Scarnati et al., 2018; Cioni et al., 2019; Heumüller et al., 2019; Maity et al., 2020). JF635HT labeling was examined 24 hours after exposing rat cortical neurons in primary culture to CPXH and protein synthesis inhibitors or suitable controls (see Materials and Methods for further details). For each imaged cell, we quantified the ratio of JF635HT fluorescence to that of mTurquoise2 fluorescence. This allowed us to limit the analysis to cells expressing the HaloTag-mTurq2 fusion protein and normalize for some variability in expression levels among such cells. Then, for each inhibitor, we quantified the fold reduction in mean JF635HT/mTurquoise2 fluorescence ratio in comparison to controls, providing a quantitative assessment of protein synthesis suppression in these preparations (Figure 1; Supplementary Figure S1). As shown in Figures 1B,D, protein synthesis inhibition was extremely effective for CHX (data regarding CHX effects and Figure 1B present previously published results shown here in sake of completeness; Cohen et al., 2020) and quite effective for ANI, resulting in ~39.2x and ~ 4.2x reductions in JF635HT labeling, respectively. Inhibition was, however, unsatisfactory for puromycin at the selected concentrations and measurement duration (~1.9x, Figure 1F). As we aimed for manipulations that effect rapid and strong suppression of protein synthesis, only CHX and ANI were used in subsequent experiments.

### Timeline of PSI-triggered cell death

**Figure 2:**
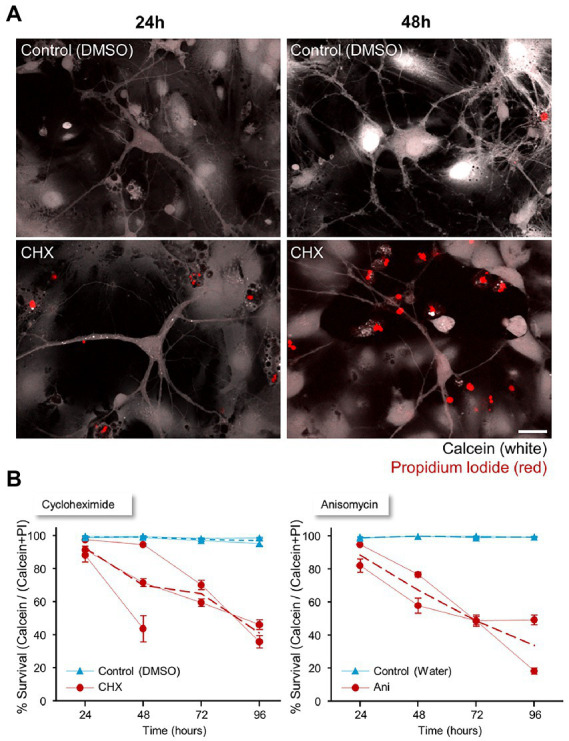
Timeline of neuronal survival following protein synthesis inhibitor treatment. **(A)** Two to 2.5 weeks after plating, cortical neurons were treated with cycloheximide (CHX), anisomycin (ANI), or carrier solution (0.1% DMSO in cell culture media, or 0.3% water) for a range of durations (24, 48, 72, or 96 hours). The cells were loaded with Calcein-AM (2 μM; 30 min), and Propidium Iodide (2.5 μM) was added immediately before imaging. The number of dead (PI-positive) cells and live (Calcein-positive) cells in each field of view was counted for each agent and time point. Scale bar 20 μm. **(B)** Cell viability was calculated as the average ratio of live (Calcein-positive) cells to total cell count (Calcein positive cells + PI positive cells). Data shown separately for each experiment (separate cell culture preparations from different rat pup litters, PSI/control treatments, labelling and analysis; CHX: 3 experiments; ANI: 2 experiments). Dashed lines are averages across experiments. Error bars are SEM of ratios measured at separate fields of view (between 13 and 59) at each time in each experiment.

The aim of this study was to expose aspects of synaptic and neuronal physiology that are particularly sensitive to interruptions in the supply of newly synthesized proteins. As such, these would be expected to manifest (long-) before overt signs of cell death appear. To obtain a better estimate of this time frame, we measured the time course of PSI-triggered cell death in cortical neurons in primary culture, using Calcein-AM and Propidium Iodide (Sadeh et al., 2015, 2016). Calcein-AM crosses the neuronal membrane, where, in viable cells, it is converted to fluorescent Calcein. In contrast, Propidium Iodide does not enter cells with intact membranes, but in dying or dead cells, whose membrane permeability is compromised, it reaches the nucleus and intercalates into the DNA. Together, these two reporters provide counts of viable and dying/dead cells. To this end, age-matched cell-culture preparations were treated with CHX or ANI or carrier solutions for 24, 48, 72, or 96 hours. Following these periods, the cells were exposed to Calcein-AM and Propidium Iodide (PI) as described in Materials and Methods and imaged immediately. Numbers of PI- and Calcein-positive cells were counted in each field of view and a measure of cell viability was calculated as the average ratio of live (Calcein-positive cells) to total cell counts (Calcein-positive cells + PI-positive cells) across all fields of view and experiments (2–3 independent experiments). As shown in Figure 2, only a small reduction in this measure of cell viability was observed after 24 h of PSI treatment, but the reduction became increasingly pronounced at 48 hours and even more so at 72 h. At 96 h, most of the cells were dead. In contrast, cells from time-matched preparations treated with carrier solutions displayed close to 100% survival at all time points. We note that the preparations used here are mixed neuron–glia cultures, and thus readouts relate to both cell types. We also note that visible changes in cell morphology hint that the Calcein-AM/Propidium Iodide method used here underestimates the actual degree of cell death, in particular at the 72- and 96-h time points. Nevertheless, these observations, as well as others described later on, suggest that most cells are still viable within the first 24 h following abrupt interruptions of protein synthesis, leading us to focus on the effects of such interruptions on synaptic and neuronal physiology during this early time window.

### Effects of PSIs on synaptic persistence

**Figure 3:**
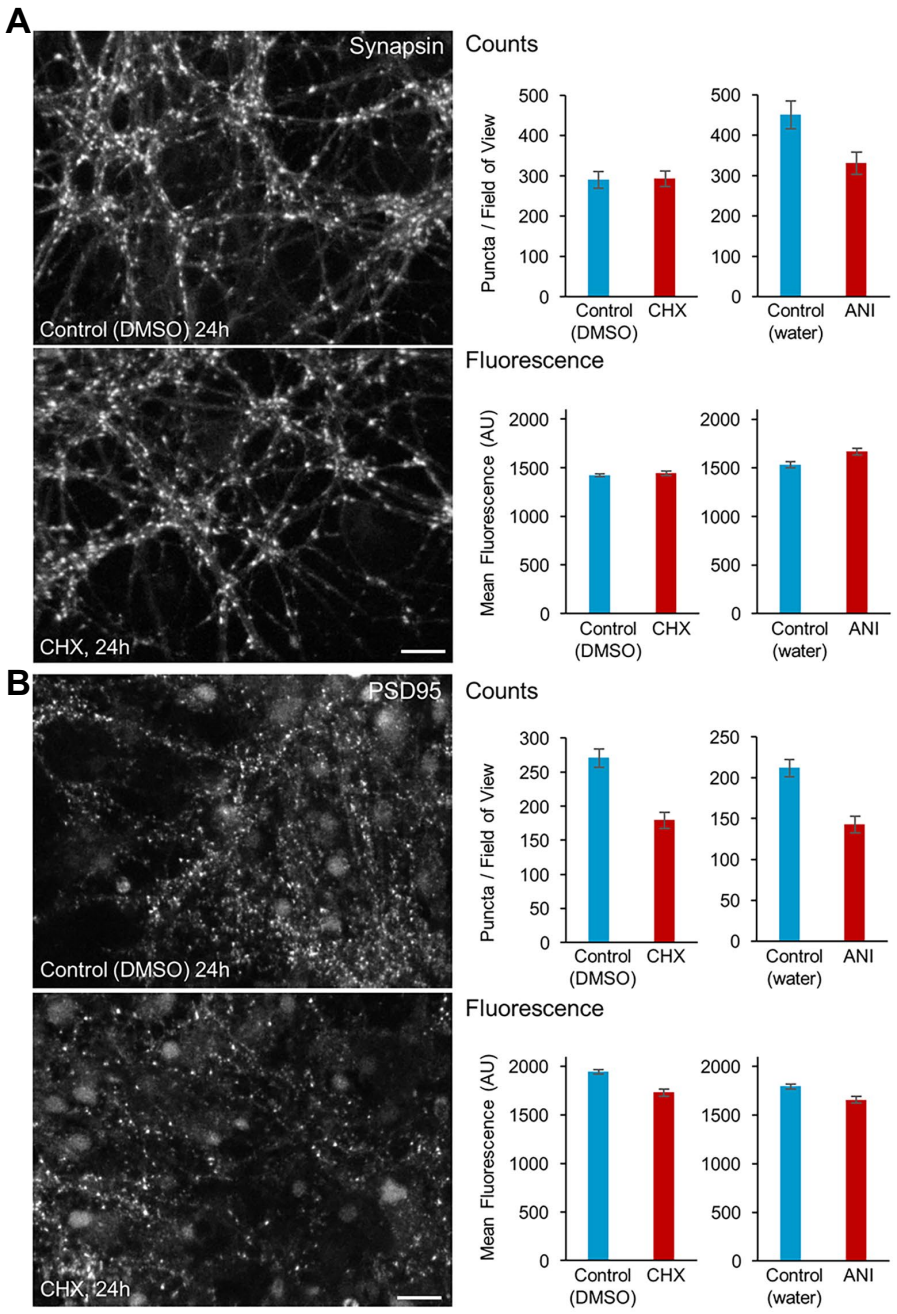
Effects of protein synthesis inhibitor treatment on synaptic persistence. Cortical neurons were grown for 2.5 weeks in cell culture wells (8 mm diameter cylinders adhered to coverslips). Matched cell culture wells (2–4 per condition) were treated with protein synthesis inhibitors or vehicle solutions for 24 h, fixed and stained with antibodies against the presynaptic protein Synapsin **(A)** and the postsynaptic protein PSD-95 **(B)**. Synapse numbers per field of view and molecular contents (mean fluorescence values) were quantified programmatically and compared. Bars: Synapsin: 10 μm, PSD-95: 20 μm. Mean ± SEM of data collected in separate fields of view. >30 fields of view from at least 2 cell culture wells per condition. See Supplementary Figure S2 for presentation by well.

We first set out to determine the degree to which acute interruptions of protein synthesis affect overall synaptic persistence. To that end, we exposed rat cortical neurons to CHX or ANI for 24 hours, fixed and stained the neurons with antibodies against the presynaptic protein Synapsin, previously shown to be a particularly reliable marker of presynaptic specializations (Micheva and Smith, 2007) and PSD-95, a major postsynaptic density component of glutamatergic synapses. Suppressing protein synthesis for 24 h led to reductions in PSD-95 puncta counts (Figure 3B), whereas for synapsin, modest reductions were observed for ANI (possibly attributable to cell culture preparation variability; Supplementary Figure S2) and none for CHX (Figure 2A). Interestingly, the average fluorescence of remaining Synapsin puncta was not reduced, whereas that of PSD-95 puncta was reduced only slightly (in agreement with previously reported changes observed after exposure to ANI for 10 hours; Cohen et al., 2013), which would be consistent with the preferential loss of smaller (dimmer) postsynaptic specializations. Thus, acute suppressions of protein synthesis for 24 hours seem to drive some synaptic loss, which appears to be more noticeable on the postsynaptic side.

### Effects of PSIs on synaptic vesicle recycling

**Figure 4:**
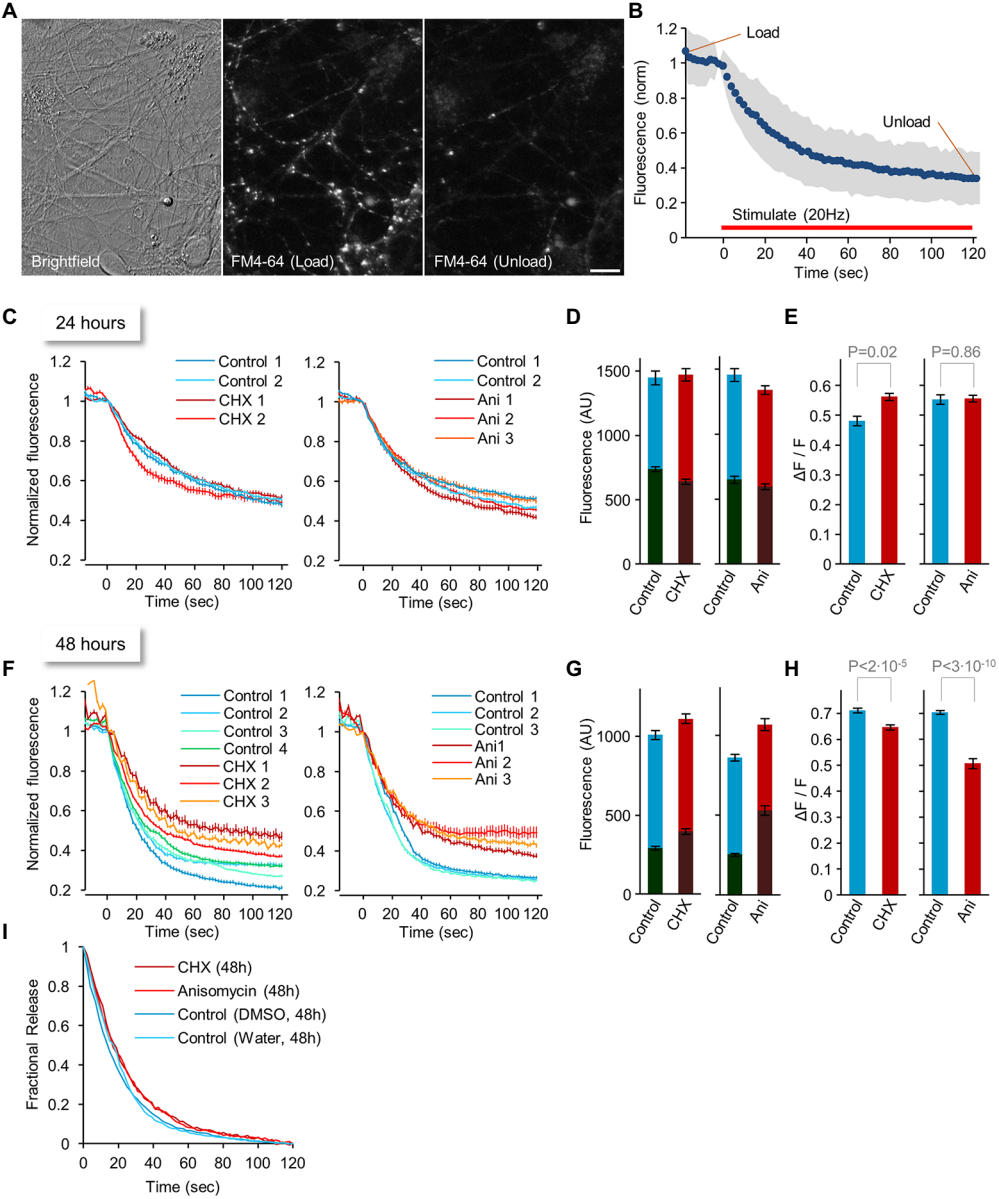
Changes in synaptic vesicle recycling after 24 or 48 hours of protein synthesis inhibition. **(A)** Images of FM4-64 labeling at structures that most likely represent functionally active presynaptic boutons (left: brightfield image, middle: FM4-64 dye after loading, right: dye after unloading). Bar, 10 μm. **(B)** Experimental procedure: 2–2.5-week-old neuronal networks were loaded with FM4-64 by field stimulation. Images as in **(A)** were obtained at several fields of view. Dye unloading was carried out by field stimulation (20 Hz, 120 sec.; red line) while imaging at 2 sec intervals at one field of view. After dye unloading was completed, a second image of all other fields of view was acquired. Dye loading and unloading (measures of endocytosis and exocytosis) were quantified for individual presynaptic boutons at each field of view by measuring the FM4-64 fluorescence of presynaptic puncta after loading, and after unloading. For fields of view imaged during the unloading procedure, the fluorescence of each bouton was normalized to its fluorescence intensity before dye unloading, and normalized data were pooled to create release curves (in blue; standard deviation is marked in gray). For all other fields of view, the fluorescence of each bouton was quantified at two time points (“Load” and “Unload”), followed by a calculation of the fraction of dye released by the unloading stimuli. **(C–E)** Changes in synaptic vesicle recycling after 24 h of protein synthesis inhibitor treatment: Dye release curves **(C)**, Absolute fluorescence after loading and unloading **(D)**, and Fractional dye release **(E)**. **(F–I)** changes in synaptic vesicle recycling after 48 hours of protein synthesis inhibitor treatment: Dye release curves **(F)**, Absolute fluorescence after loading and unloading **(G)**, Fractional dye release **(H)**, and Release kinetics **(I)**. Values in **(E,H)** indicate *p* values from t-test assuming unequal variances. Each curve in **(C,F)** indicates average ± SEM of individual boutons in single fields of view collected in separate experiments. Each curve was generated from ~200 boutons on average. Bars in **(D–H)** indicate Average ± SEM of regions of interest, from all imaged fields of view. Data in **(D,E)** are from >12 fields of view and ~5,400 boutons on average per condition. Data in **(G,H)** are from >19 fields of view and ~5,000 boutons on average per condition.

Synaptic function strongly depends on intact exocytosis and endocytosis of presynaptic vesicles, complex dynamic processes that depend on myriad specific molecules, including relatively short-lived yet critical ones such as Munc-13 (Cohen and Ziv, 2019). Indeed, prior studies have shown that short (1–3 h) PSI exposure can alter neurotransmitter release and vesicle replenishment (Bradley and Sporns, 1999; Ma et al., 1999; Scarnati et al., 2018). To measure the effects of longer exposures to PSIs on neurotransmitter release and vesicle recycling, we exposed neurons in culture to CHX or ANI or carrier solutions (DMSO and water, respectively) for hours and days and measured synaptic vesicle recycling using the vital dye FM4-64 and field stimulation. FM4-64 dye intercalates into cell membranes and becomes internalized in recycling synaptic vesicles, staining them brightly until these are exocytosed and re-exposed to the external solution. To that end, cortical neurons raised in culture for 2 to 2.5 weeks and exposed to PSIs or carrier solutions for various durations, were placed in a perfusion chamber equipped for field stimulation and labeled with FM4-64 in a physiological solution (Tyrode’s; see Materials and Methods for further details). Briefly, labeling (‘loading’) was carried out by stimulating the neurons in the presence of FM4-64 at 20 Hz for 60 sec, waiting for 30 sec, and then vigorously washing off excess dye with Tyrode’s solution for 60 sec. Images of labeled presynaptic boutons were then obtained at several fields of view as shown in Figure 4A. Dye unloading was then carried out by stimulating the neurons at 20 Hz for 120 sec while imaging at 2 sec intervals at one field of view. After dye unloading was completed, a second image was acquired at all other fields of view. Dye loading and unloading (measures of endocytosis and exocytosis) were quantified for individual presynaptic boutons at each field of view by measuring the FM4-64 fluorescence of presynaptic puncta after loading and after unloading. For fields of view imaged during the unloading procedure, the fluorescence of each bouton was normalized to its fluorescence before dye unloading, and normalized data were pooled to create release curves as in Figure 4B. For all other fields of view, fluorescence of each bouton was quantified at two time points as illustrated in Figure 4B, followed by a calculation of the fraction of dye released by the unloading stimuli.

In initial experiments carried out following 2, 4, 7 and 9 hours of PSI (CHX) exposure, we observed no obvious changes in dye release kinetics (unpublished observations). We therefore repeated the experiments following longer exposures: 24, 48 (and 72) hours. Somewhat surprisingly, 24 hours in the presence of either CHX or ANI had very little effect on presynaptic vesicle recycling, as assessed by comparing dye release curves (Figure 4C), absolute fluorescence after loading and unloading (Figure 4D) or fractional dye release (Figure 4E) to suitable controls. After 48 hours, however, differences became noticeable (Figures 4F–H). Although raw fluorescence measurements were suggestive of elevated levels of non-specific labeling (Figure 4G), correcting for these effects indicated that fractional dye release was reduced (Figure 4H), as were release kinetics (Figure 4I). Interestingly, even after 72 hours of PSI exposure, and despite widespread cell death, we still found fields of view in which synapses exhibited surprisingly robust dye loading and unloading (one example is shown in Supplementary Figure S3).

For the 24- and 48-hour time points, we also measured the fraction of FM4-64 labeled puncta that, once labeled, grew dimmer following a second stimulus train (that is, responded to the unloading procedure). We first measured the reduction in fluorescence at labeled but non-punctate objects to assess dimming due to dye washout and photobleaching, finding that it was in the range of 8 to 12%. Consequently, fluorescence reductions greater than 15% were considered to reflect responses to unloading stimuli. This analysis revealed no major effects of PSI treatment on the fraction of responding boutons (24 hours: 92 and 95% responders, control vs. CHX respectively; 96 and 97% responders, control vs. ANI, respectively; 48 hours: 99 and 99% responders, control vs. CHX respectively; 99 and 95% responders, control vs. ANI, respectively). The lack of strong effects in these analyses is not surprising, perhaps, given that dye loading (i.e., labeling) depends on responsiveness to stimuli as well. It is worth noting, however, that whereas absolute numbers of FM4-64 labeled puncta at 24 hours were comparable at all conditions, after 48 hours in the presence of PSIs numbers of labeled puncta were significantly lower in comparison to controls (~60% CHX; ~63% ANI of respective controls). These reductions are in general agreement with the considerable cell death measured at this time point (Figure 2).

Taken together, these data indicate that abrupt interruptions in the supply of newly synthesized protein copies impact evoked synaptic vesicle release and recycling at relatively slow time scales (at least as far as the assays used here were able to determine), that roughly track the time course of cell death in the same preparations.

### Effects of PSIs on spontaneous network activity

**Figure 5:**
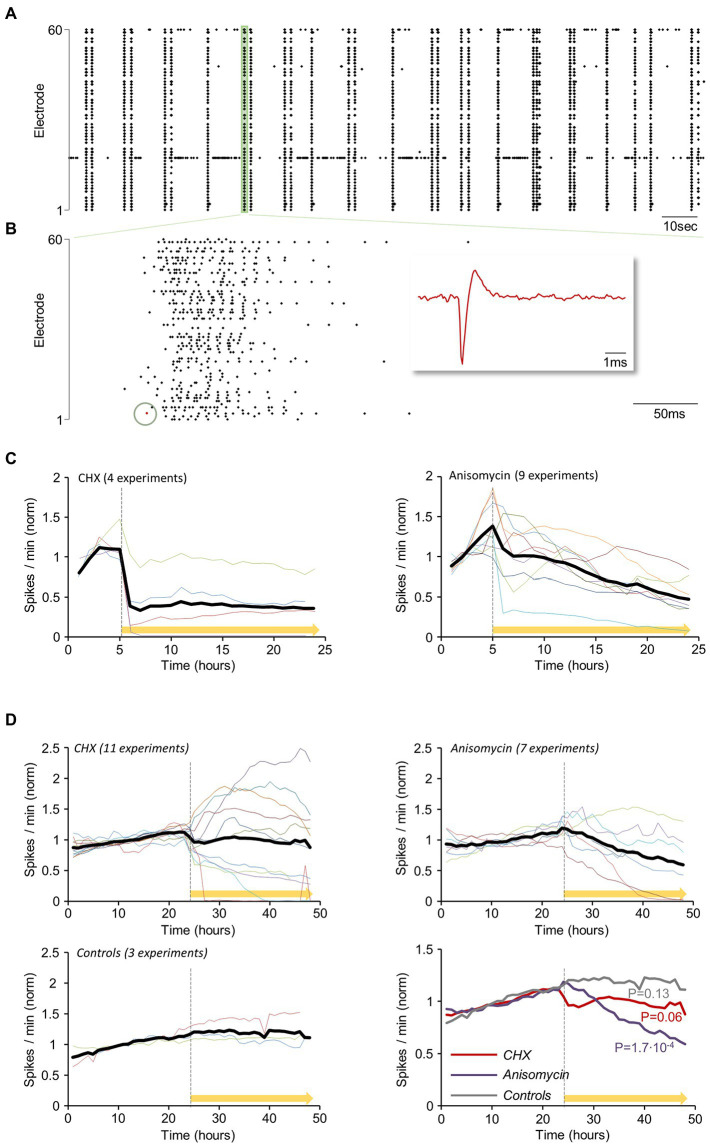
Reductions in spontaneous network activity levels within hours of protein synthesis inhibition. Rat cortical neurons were grown on multielectrode array (MEA) substrates for 2.5–3.5 weeks. The preparations were then mounted on an MEA amplifier and action potentials (APs) were recorded from the 59 MEA electrodes. **(A)** Spontaneous network activity measured by MEA recordings: Action potentials (APs, Spikes) recorded from all electrodes (3-min excerpt of a recording). **(B)** Higher magnification of the region highlighted in green at **(A)**. Each dot corresponds to a threshold crossing (representing an AP fired and recorded at one of the electrodes, as shown in magnified view in the red inset, matching the red dot in the recording excerpt). **(C)** The spontaneous activity was measured for 5 hours, after which cycloheximide (CHX: *n* = 4) or anisomycin (ANI: *n* = 9) was added to the MEA dish (yellow bar). Recordings were then continued for another 16 hours. APs measured from all electrodes were accumulated at 1 min intervals, and normalized to mean spike rates during the first 3 hours of each experiment. Colored lines represent individual experiments (different MEAs, mostly from different cell culture preparations). Thick black line is the average of all experiments. **(D)** Experiments as in **(C)**, but with FIGURE 5 (Continued)continual perfusion with fresh growth media at very slow rates (~1–2 volumes per day). The networks were perfused for periods of 24–48 h, after which spontaneous activity levels were recorded for an additional 24 h (baseline). At this point, CHX (*n* = 11), ANI (*n* = 7), or carrier solutions (DMSO/water: *n* = 3) were added to the dish and the perfusion media, and network activity was recorded for another 24 h. Activity levels were normalized to the mean of the first 24 hours. A comparison of all three conditions is shown in the bottom right panel. *p* values shown here were calculated by separately fitting lines (by linear regression) to each 24-hour period (before and after exposure to CHX/ANI/Carrier solution) and for each trace (experiment), and then comparing the slopes of these lines by two-sided paired t-tests.

Networks of cortical neurons in culture exhibit spontaneous activity which usually takes the form of network-wide bursts of action potentials (e.g., Maeda et al., 1995; van Pelt et al., 2004; Eytan and Marom, 2006; Wagenaar et al., 2006; Kaufman et al., 2014). Spontaneous activity levels represent a sensitive measure of neuronal viability as they are strongly affected by ion gradients, channel states and numbers, synaptic functionality and many other physiological parameters. To examine how acute suppressions of protein synthesis affect spontaneous network activity levels, we grew rat cortical neurons on multielectrode array (MEA) substrates and recorded action potentials (APs) from the MEAs 59 electrodes as described previously (Minerbi et al., 2009; Hazan and Ziv, 2017). Spontaneous activity (as shown for example in Figures 5A,B) was measured for 5 hours, after which CHX or ANI were applied. Recordings were then continued for another 16 hours. APs measured from all electrodes were accumulated at 1 min intervals and then normalized to reference periods (the first 3 hours of each experiment) to correct for differences in baseline activity levels. In all experiments (Figure 5C, CHX: *n* = 4; ANI: *n* = 9) PSI application was followed by substantial decreases in network activity levels (see also Sharma et al., 2012; Greenberg et al., 2014). Interestingly, CHX led to almost instantaneous reductions followed by a plateau, whereas ANI led to more gradual reductions over time.

We (e.g., Minerbi et al., 2009; Kaufman et al., 2014; Rubinski and Ziv, 2015; Hazan and Ziv, 2020) and others (Saalfrank et al., 2015) have found that cell culture viability and spontaneous activity levels are greatly enhanced by continual, slow perfusion (~1–2 volumes/day) with fresh growth media. We thus examined how PSIs affect spontaneous activity levels in networks growing in such conditions. To that end, networks of cortical neurons grown on MEA dishes were perfused for 24–48 hours, after which spontaneous activity levels were recorded for an additional 24 hours (baseline). At this point, CHX, ANI or carrier solutions (DMSO/water) were added to the dish and the perfusion media, and activity was recorded for another 24 hours. Activity levels were normalized to the mean of the first 24 hours. In these experiments, the effects PSIs had on spontaneous activity levels were more variable (Figure 5D). While ANI typically led to gradual reductions in activity levels, CHX drove rapid changes in both directions, at least initially. Nevertheless, the average effect was similar to that observed in non-perfused preparations, namely, an instantaneous reduction, followed by a plateau of lower activity levels. The reason for this variability is unclear, but as described later, we suspect this might relate to differential effects on excitatory and inhibitory synapses (or neurons). In contrast, no effects were observed following the introduction of carrier solutions (Figure 5D). These experiments suggest that PSIs tend to suppress spontaneous network activity, but effects differ by inhibitor and are sensitive to experimental conditions.

### Effects of PSIs on synaptic tenacity and survival

**Figure 6:**
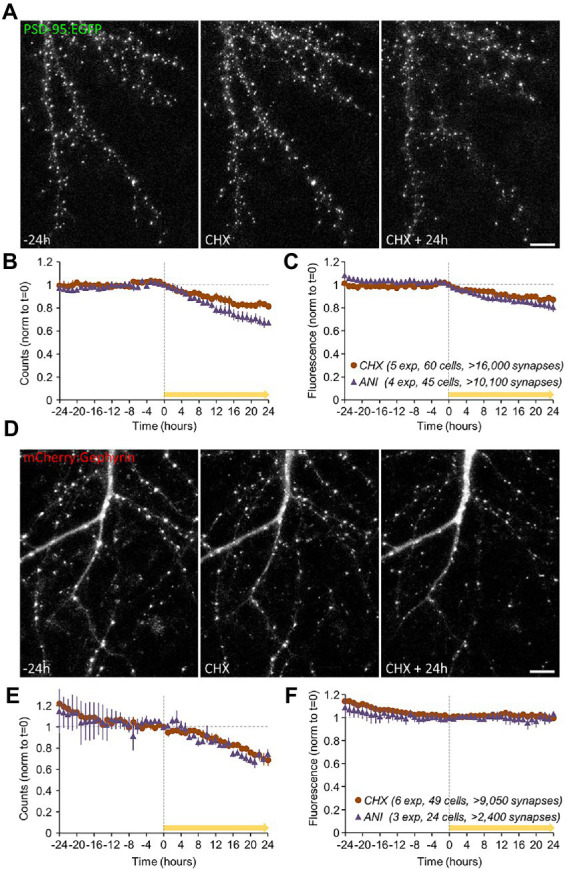
Effects of PSIs on synaptic survival. Synapses of neurons expressing fluorescently tagged variants of PSD-95 (PSD-95:EGFP); data in **(A–C)** and Gephyrin (mCherry:Gephyrin); data in **(D–F)** were imaged at 1-hour intervals by confocal microscopy. The synapses were followed under baseline conditions for 24 hours after which the neurons were exposed to CHX, ANI, or carrier solutions (DMSO/water) and followed for an additional 24 hours (yellow arrows). After the experiments, the counts and fluorescence of automatically detected punctate objects were measured (values were normalized to the respective values at *t* = 0 on a per-cell basis as detailed in Main text and Methods). **(A)** Synapses of neurons expressing PSD-95:EGFP, imaged by confocal microscopy. **(B)** Changes in PSD-95:EGFP puncta counts following PSI addition (CHX/ANI added from *t* = 0; yellow bar). **(C)** Reductions in the fluorescence of the remaining puncta following PSI application. Data in **(B,C)** are from: 5 experiments (mostly from separate cell culture preparations), 60 cells, >16,000 synapses (CHX), and: 4 experiments, 45 cells, >10,100 synapses (ANI). **(D)** Synapses of neurons expressing mCherry:Gephyrin, imaged by confocal microscopy. **(E)** Changes in mCherry:Gephyrin puncta counts (small reductions were observed during the baseline periods; CHX/ANI added from t = 0). **(F)** mCherry:Gephyrin puncta fluorescence. Scale bars, 10 μm. Error bars represent ±SEM of experiments. Data in **(E,F)** are from: 6 experiments, 49 cells, >9,050 synapses (CHX), and: 3 experiments, 24 cells, >2,400 synapses (ANI).

The experiments of Figure 3 revealed that 24-hour exposures to PSIs led to some loss of postsynaptic specializations, but the time course of this loss was not determined. Moreover, as PSD-95 is an excitatory synapse postsynaptic protein, effects on inhibitory synapses remained unknown. Finally, immunocytochemical analysis carried out in separate preparations is incapable of providing information on the tenacity of specific synapses, i.e., their capacity to preserve their properties over time. To address these questions we carried out live imaging of neurons expressing fluorescently tagged variants of PSD-95 (PSD-95:EGFP; Minerbi et al., 2009) and Gephyrin (mCherry:Gephyrin), the latter being a major postsynaptic density protein of GABAergic (and glycinergic) synapses (Tyagarajan and Fritschy, 2014) which has been used extensively for longitudinal imaging of inhibitory synapses (e.g., Hanus et al., 2006; Dobie and Craig, 2011; Chen et al., 2012; van Versendaal et al., 2012; Vlachos et al., 2013; Isshiki et al., 2014; Rubinski and Ziv, 2015; Villa et al., 2016). Specifically, PSD-95:EGFP and mCherry:Gephyrin were expressed in cortical neurons in primary culture using lentiviral vectors, and synapses expressing these fusion proteins were imaged by confocal microscopy at one-hour intervals (Figures 6A,D) under conditions of continuous perfusion as mentioned above and described previously (e.g., Minerbi et al., 2009; Rubinski and Ziv, 2015; Hazan and Ziv, 2020; see Materials and Methods for further details). Synapses were followed under baseline conditions for 24 hours, after which the neurons were exposed to CHX, ANI or carrier solutions (DMSO/water) and followed for an additional 24 hours. After the experiments, maximal intensity projections of all Z-sections were calculated, punctate objects were detected automatically, and their counts and fluorescence were measured. To compensate for variability in reporter expression levels and to allow for data pooling, the fluorescence measured at each synapse was normalized to the average puncta fluorescence measured for its respective neuron at *t* = 24 h.

In agreement with the data shown in Figure 3, PSIs led to substantial reductions in PSD-95:EGFP puncta counts (Figure 6B) and a modest reduction in the fluorescence of the remaining puncta (Figure 6C). The latter observation is also evident in the leftward shift of PSD-95:EGFP puncta fluorescence distributions (Supplementary Figures S4A,B). These findings indicate that PSIs reduced the numbers (and possibly the sizes) of excitatory synapse postsynaptic specializations.

A similar trend was observed for mCherry:Gephyrin puncta counts (Figure 6E), even on the background of slight reductions in mCherry:Gephyrin counts during baseline periods (see also Rubinski and Ziv, 2015). In contrast to PSD-95:EGFP, however, unequivocal changes in mCherry:Gephyrin puncta fluorescence were not observed (Figure 6F), although we found hints to similar trends in changes to mCherry:Gephyrin puncta fluorescence distributions (Supplementary Figures S4C,D).

As these experiments were carried out in a combined imaging/MEA recording system (Minerbi et al., 2009; Rubinski and Ziv, 2015; Hazan and Ziv, 2020) we could examine the degree to which changes in excitatory and inhibitory synapse numbers 24 hours post treatment correlated with changes in spontaneous activity levels (as in Figure 5D) on a network to network basis. As shown in Supplementary Figure S5, we observed that the extent of excitatory synapse loss correlated positively (*r* = 0.73) with reductions in activity levels, whereas the extent of inhibitory synapse loss correlated negatively (*r* = −0.43) with reductions in activity levels (as might be expected). These observations thus provide a possible explanation to the divergent effects of CHX on the spontaneous activity levels shown in Figure 5D.

**Figure 7:**
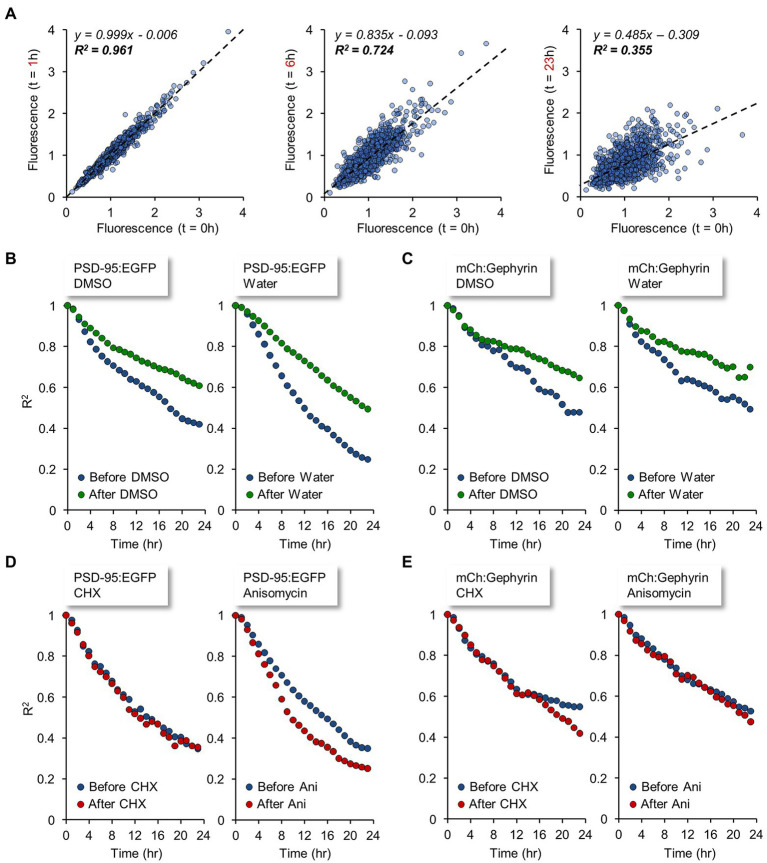
Synaptic tenacity is impaired by interruptions in the supply of newly synthesized proteins. Individual PSD-95:EGFP and mCherry:Gephyrin puncta were tracked over time and the normalized fluorescence of each punctum was compared to its normalized fluorescence at the start of each 24 hour window: the start of the baseline recording, and time of PSI (CHX/DMSO) or carrier solution (DMSO/water) addition, both denoted by *t* = 0. **(A)** The normalized fluorescence values of each synapse at time *t* = 1, *t* = 6, *t* = 23 hours are plotted relative to the normalized fluorescence values at *t* = 0 (in this dataset, the time of CHX addition). Dashed lines indicate linear regression fits. Equations of the fits and coefficient of determination (R^2^), signifying the goodness of fit with the original values, are indicated in each plot. Data are from PSD-95:EGFP puncta (1,051 synapses). Linear fits at later times typically display lower R-squared (goodness-of-fit) values as well as shallower slopes. **(B–E)** The coefficient of determination (R^2^) is plotted as a function of the time interval, providing a measure of the rates of synaptic configuration change. **(B)** PSD-95:EGFP before (blue) and after (green) exposure to carrier solutions. **(C)** mCherry:Gephyrin before (blue) and after (green) exposure to carrier solutions. **(D)** PSD-95:EGFP before (blue) and after (red) exposure to PSIs. **(E)** mCherry:Gephyrin before (blue) and after (red) exposure to PSIs. Data from same experiments shown in Figure 6.

Interestingly, changes in synaptic counts (and reporter fluorescence, where observed) became noticeable within 4 to 8 hours of PSI exposure (Figures 6B,C,E), suggesting that synaptic maintenance and preservation of synaptic properties can be sensitive to interruptions in the supply of newly synthesized proteins within this short time frame. To better measure this phenomenon, we tracked individual PSD-95:EGFP and mCherry:Gephyrin puncta over time, and compared their normalized fluorescence to their normalized fluorescence at the beginning of each 24-hour time window (before and after exposure to PSIs). Specifically, normalized fluorescence values of each synapse were plotted at increasing time intervals against the fluorescence values of the same synapses at the beginning of the time window, and the goodness of fit with original values was estimated by linear regression as shown in Figure 7A. The coefficient of determination (R^2^) was then plotted as a function of the time interval (Figures 7B–E; see also Statman et al., 2014; Rubinski and Ziv, 2015; Ziv and Brenner, 2018; Hazan and Ziv, 2020), providing a measure of the rate at which individual synapses deviated from their initial ‘sizes’. This, in other words, provided a measure of the degree to which synaptic configurations were conserved.

As shown in Figures 7B,C, under baseline conditions and exposure to carrier solutions, the rates of synaptic configuration change were considerable. In line with a previous study (Rubinski and Ziv, 2015), this phenomenon was more evident for excitatory, compared to inhibitory, synapses. Change rates for both classes of synapses (excitatory and inhibitory) tended to slow down a little as experiments progressed, pointing to some non-stationarity in the experimental conditions (see also Minerbi et al., 2009). In contrast to the moderate slowdown of change rates observed in the control/carrier solutions, exposure to PSIs resulted in similar (~unchanged) or faster rates of synaptic configuration change (Figures 7D,E). Thus, in comparison to carrier solutions, PSIs seemed to accelerate the rate of synaptic configuration change, indicating that these impaired the capacity of individual synapses to maintain their individual properties. Here too, effects were observable within several hours of PSI exposure, suggesting that synaptic tenacity is impaired by interruptions in the supply of newly synthesized proteins within this time frame.

### Effects of PSIs on energy metabolism

**Figure 8:**
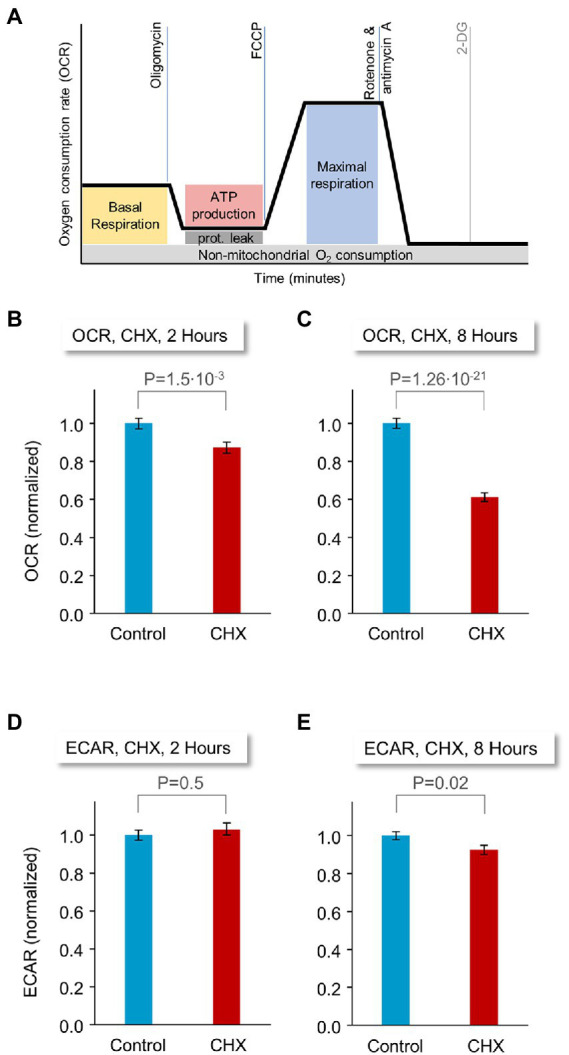
Effects of PSI exposure on energy production capacity. Rat cortical neurons were plated on application-specific 96-well microplates. 18–21 days after plating, the neurons were treated with CHX or carrier solution (DMSO), for 2 or 8 hours. Oxygen consumption rates (OCR) and extracellular acidification rates (ECAR), measures of mitochondrial respiration and glycolysis, respectively, were then monitored in a Seahorse flux analyzer, following the procedure illustrated in **(A)**. **(B)** Baseline OCR values. Preparations were treated for 2 h with CHX or carrier solution (DMSO). 120 wells for each condition from 4 separate experiments (separate plates, each from a different cell culture preparation), each normalized to the average OCR of control (DMSO-treated) wells of each experiment (non-normalized data shown for each experiment separately in Supplementary Figure S6A). **(C)** Baseline OCR values. Preparations were treated for 8 h with CHX or carrier solution (DMSO). 90 wells for each condition from 3 separate experiments (see also Supplementary Figure S6B). **(D)** Baseline ECAR, 2 h CHX or carrier solution. **(E)** Baseline ECAR, 8 h CHX or carrier solution (see also Supplementary Figure S6C,D). Same experiments as in **(A,B)**. *p*-values are from two-sided t-tests assuming unequal variances. Error bars indicate ±SEM of microwells.

The manifestation of observable changes in network activity, synaptic persistence and tenacity within a few hours of PSI treatment raised the possibility that these changes might reflect (directly or indirectly) rapid changes in the energy production capacity of PSI treated cells. To measure the impact of acute suppression of protein synthesis on mitochondrial respiration and glycolysis in these preparations, we compared oxygen consumption rates (OCR) and extracellular acidification rates (ECAR), respectively, in CHX-treated and untreated rat cortical neurons. Specifically, neurons were grown in application-specific 96-well cell culture microplates for 18–21 days, exposed to CHX or carrier solution (DMSO) for 2 or 8 hours, mounted in a Seahorse flux analyzer and subjected to sequential pharmacological manipulations (illustrated in Figure 8A) that profile energy metabolism and mitochondrial respiration [oligomycin, carbonyl cyanide 4-(trifluoromethoxy) phenylhydrazone (FCCP), rotenone/antimycin A (Rot/AA) and 2-deoxyglucose (2-DG); see Materials and Methods for details on calibration and assay specifics].

Strikingly, we observed nearly two-fold differences in baseline oxygen consumption rates (Figure 8A, yellow box) between preparations treated for 8 hours with CHX or carrier solution (DMSO; Figure 8C; 90 wells for each condition from 3 separate experiments). This trend was already observable after 2 hours of PSI treatment (Figure 8B, 120 wells for each condition, 4 separate experiments). In contrast, effects on glycolysis were insignificant, for the most part (Figures 8D,E), and, where statistically significant, the effect was attributable to a single replicate (Supplementary Figure S6D, data shown separately for each experiment).

Energy metabolism profiling using sequential pharmacological manipulations (inhibiting the mitochondrial ATP synthase; allowing free transport of hydrogen ions across the mitochondrial membrane; poisoning the mitochondrial electron transport chain) using the agents described above in the manner illustrated in Figure 8A, revealed significant impairments in mitochondrial function when OCR values were normalized to baseline levels (3 experiments, 8 hours of exposure to CHX; Supplementary Figure S6E). Conversely, inhibiting glycolysis failed to uncover differences between CHX treated and untreated cells (Supplementary Figure S6F). Collectively, these findings point to the sensitivity of mitochondrial-based oxidative phosphorylation (but not glycolysis) to abrupt suppression of protein synthesis. It should be noted that some protein synthesis occurs directly in mitochondria, but CHX is not an effective inhibitor of mitochondrial protein synthesis at the concentrations used here (Beattie et al., 1967; Küntzel, 1969; Lietman, 1971; Ibrahim and Beattie, 1973).

### Identification of rapidly lost proteins – A proteomic analysis

**Figure 9:**
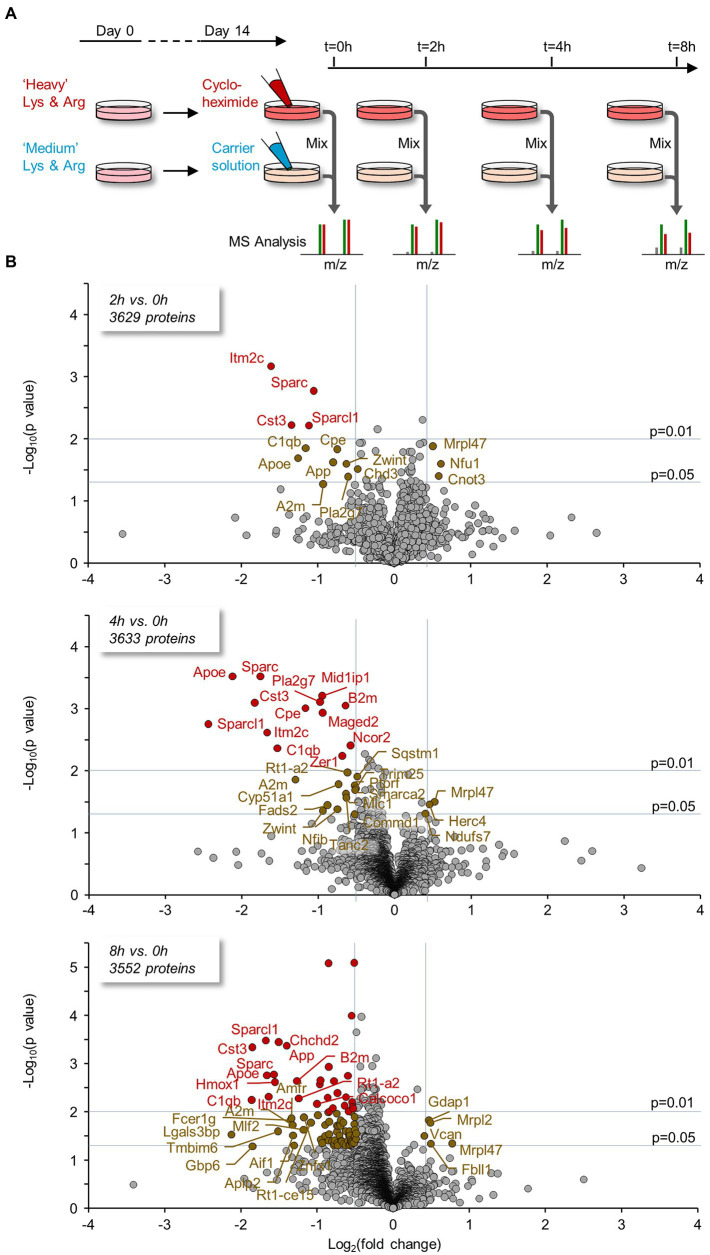
Proteins lost rapidly following protein synthesis suppression identified using SILAC and mass spectrometry. **(A)** Cortical neurons were grown for 14 days in media containing discernible, isotopically labeled amino acids (“Heavy” variants: Lys8 - ^13^C_6_, ^15^N_2_; Arg10 - ^13^C_6_, ^15^N_4_, or “Medium” variants: Lys6 - ^13^C_6_; Arg6 - ^13^C_6_). At this point, CHX or a carrier solution was added; the cells were then harvested 0, 2, 4, or 8 hours after exposure. Time-matched pairs were mixed and the samples were analyzed by mass spectrometry (see Materials and Methods for details). For each protein at each FIGURE 9 (Continued) time point a ‘H/M ratio’ was obtained; such ratios provide an estimate of the residual amounts of each protein following PSI exposure relative to the controls. Five experiments (separate cell culture preparations, labeling, extraction, and MS analysis), each with 4 time points, were performed. A minimum of 5 peptide pairs from at least 3 experiments was required for an estimate to be included in subsequent analyses. **(B)** Proteins for which significant changes occurred were detected by plotting the log_2_ fold change (FC) at 2, 4, and 8 hours compared to *t* = 0 against the statistical significance of the change (‘volcano plots’). ‘Biologically significant’ fold-changes were considered as such if they were greater than two standard deviations of population log_2_ ratios at *t* = 0 (approximately 30%), whereas statistically significant changes were considered as such if they passed a t-test for the biological repeats with a p value ≤0.05 (see Materials and Methods). Two proteins were excluded here for improved visibility (large FC but *p* ≫ 0.05): 2 hours – Hacd3, log_2_FC = −11.9; 4 hours – Ndufs1, log_2_FC = −6.8.

The relatively rapid effects of PSIs on spontaneous activity, numbers and contents of postsynaptic specializations as well as mitochondrial-based oxidative phosphorylation, pointed to the importance of continuous new protein supply for maintaining vital neuronal functions. Given that turnover rates of most neuronal proteins are relatively slow (half-lives of many days), we hypothesized that these effects, observed over time scales of hours, reflected the loss of a small number of relatively important (short-lived?) proteins. To identify potentially essential early-lost proteins, we used dynamic SILAC and Mass Spectrometry as illustrated in Figure 9A. Specifically, we grew cortical neurons in culture for 14 days in media containing discernible isotopically labeled lysine and arginine, either ‘Heavy’ (H) variants: Lys8 - ^13^C_6_, ^15^N_2_; Arg10 - ^13^C_6_, ^15^N_4_, or ‘Medium’ (M) variants: Lys6 - ^13^C_6_; Arg6 - ^13^C_6_. At this point, either CHX or a carrier solution was added to the cells labeled with either set of labeled amino acids (for simplicity, we refer to cells exposed to CHX as H-labeled and to controls as M-labeled, even though in practice the labels were used interchangeably). 0, 2, 4, or 8 hours after exposure, the cells were harvested, time-matched extracts were mixed and the samples were analyzed by mass spectrometry (Figure 9A; see Materials and Methods for further details). For each protein at each time point, a ‘H/M ratio’ was obtained, based on amounts of differentially labeled but otherwise identical peptides identified in the mixed cell extracts. Such H/M ratios provide estimates of the residual amounts of each protein following PSI exposure, relative to the amount of the same protein in time-matched controls. In total, 5 separate experiments were performed, each with four time points. Given the modest changes expected over these short time scales, we enforced stringent criteria on data inclusion, accepting only measurements based on a minimum of 5 peptide pairs from at least 3 experiments (for each protein at each time point). These rather strict criteria were based on analyses of estimate variability as a function of peptides used to generate these estimates (see Supplementary Figures S7A,B for details). Nevertheless, and as shown in Supplementary Figures S7C,D, for the vast majority of proteins, estimates were based on all 5 experiments (separate cell culture preparations from different rat pup litters, labeling, extraction, and MS analysis) and more than 10 peptides per protein at each time point. To correct for slight differences in the relative amounts of material collected from treated and control sets, we normalized all H/M (experiment/control) ratios to H/M ratios measured for a group of seven abundant and particularly long-lived proteins (Toyama et al., 2013; Table 1). All H/M ratios mentioned hereafter reflect H/M ratios normalized in this fashion.

**Table 1:** Abundant long-lived proteins used for normalization.

**Protein**	**t**_**1/2**_**(days) from** **Cohen et al., 2013**
Vcan/ Cspg2	9.64
Lamin-B1	13.7
Lamin-B2	16.1
Nup155	9.37
Nup205	14.09
Macro-H2A.1	10.17
Macro-H2A.2	10.62

The list is based on Toyama et al. (2013); half-lives for the same proteins obtained in networks of cortical neurons grown in culture are from Cohen et al. (2013).

Proteins for which significant changes occurred were detected by plotting the log_2_ fold-change at 2, 4, and 8 hours compared to *t* = 0 against the statistical significance of the change (‘volcano plots’, Figure 9B; Supplementary Table S1). Biologically interesting fold-changes were considered as such if they were greater than two standard deviations of population log_2_ ratios at *t* = 0 (reductions by ~30%; the rational being that at *t* = 0, H/M ratios that differed from 1.0 almost certainly resulted from measurement noise), whereas statistically significant changes were considered as such if they passed a t-test for the biological repeats with a *p* value ≤0.05 (see Materials and Methods for details).

As shown in Figure 9B, the number of proteins with significant changes increased with time in CHX. Among these were proteins previously implicated in key cellular functions and pathways. Many of these seemed to belong (non-exclusively) to four particular groups: Regulators of synapse stability/organization (Sparc, Sparcl1, C1qb, Cst3, Cpe, Aif1, Mov10); proteins with roles in fatty acid/lipid metabolism (Fads2, Pla2g7, Apoe, App, Cyp51a1, Ormdl3, Mid1ip1, Mov10); proteins related to cell bioenergetics (Hk2, Slc25a13, and Ndufs7, the latter having increased following PSI exposure); and proteins that are implicated in Alzheimer’s disease (AD) pathology or colocalize with Aβ (App, Apoe, A2m, C1qb, Sparc, Cst3, Itm2c). Several of these proteins decreased consistently with increased exposure to the PSI. For a list of these proteins and relevant literature, please refer to Supplementary Table S2A. As noted above, the preparations used here were mixed neuron–glia cultures, and thus, it remains possible that some of these differences might have been attributable to proteins expressed by glia.

**Figure 10:**
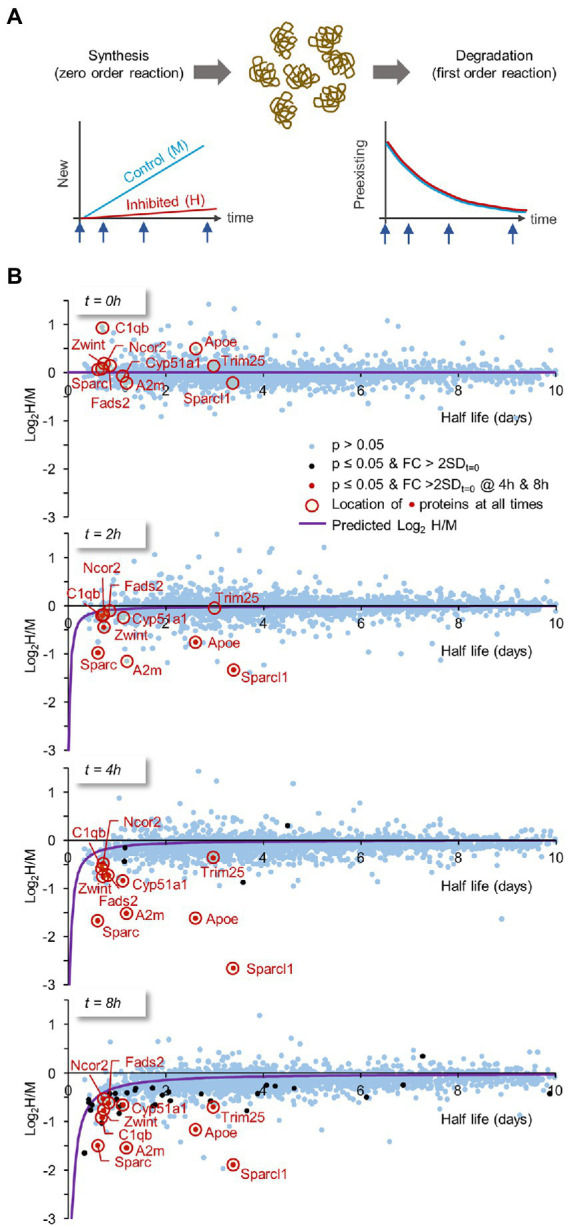
Expected dependence of log_2_H/M ratios on protein half-life times. The loss of certain proteins following PSI exposure might be expected if their turnover rates are especially high (and vice versa). Thus, we formulated a model describing the expected dependence of log_2_H/M on protein half-life times, which would then allow us to compare for each protein, its measured relative to predicted changes. **(A)** Model assumptions: two (simplified) assumptions regarding protein synthesis and degradation underlie the model: (1) protein synthesis occurs as a zero-order reaction (i.e., at a fixed rate). (2) protein degradation occurs as a first order reaction (i.e., the amount of protein lost per unit time depends on the momentary concentration of that particular protein). PSIs were considered to suppress protein synthesis rate by some factor (estimated here by the measured fold-change reduction in protein synthesis rates following CHX exposure; Figure 1), while leaving protein degradation rates intact (further details and model equations are provided in the Materials and Methods section). **(B)** Using this model we plotted, at all four time points (*t* = 0, 2, 4 and 8 hours), the measured log_2_H/M ratio for each protein against the protein’s known half life (data on half-life times from Cohen et al., 2013; Hakim et al., 2016). FIGURE 10 (Continued) Blue dots are proteins for which the change relative to *t* = 0 (Figure 9) was not statistically significant (i.e., *p* > 0.05). Black dots represent proteins for which statistically significant changes were observed and the change was greater than 2 standard deviations measured at *t* = 0. Red dots are for proteins in which statistically significant changes and above-threshold fold changes were observed at both 4- and 8-h time points. These proteins are highlighted at all time points with a red circle. The expected log_2_H/M values as a function of a protein’s half-life were plotted as purple curves. Only proteins with t_1/2_ ≤ 10 days are shown.

The loss of certain proteins following protein synthesis suppression might be expected if their turnover rates are particularly high. We consequently asked how well PSI-induced protein loss compares, on a protein-to protein basis, with known half-lives for the same proteins. To that end, we formulated a model to describe the expected dependence of log_2_(H/M) values on protein half-life. The (undoubtedly simplified) underlying assumptions (illustrated in Figure 10A) were (1) that protein synthesis occurs as a zero-order reaction (i.e., occurs at a fixed rate), whereas (2) protein degradation occurs as a first order reaction (i.e., the amount of protein lost per unit time depends on the momentary concentration of the protein in question). PSIs were considered to suppress protein synthesis rate by some factor while leaving protein degradation rates intact. Using this model (described in Materials and Methods and Table 2 therein) we compared, at all 4 time points, the measured log_2_(H/M) values for each protein to the expected values predicted by the model from the known half-life of the same proteins (Supplementary Table S3, column B; Data on half lifetimes were obtained from Cohen et al., 2013; Hakim et al., 2016) and the measured fold-change reduction in protein synthesis rates in the presence of CHX (Figure 1). As shown in Figure 10B, the overall fit with the model was good (see Materials and methods for data on the goodness of fit), even for the vast population of proteins for which changes in protein abundance did not cross the threshold of statistical significance defined here (this is particularly evident for short-lived proteins in the 4- and 8-hour plots).

**Table 2:** Symbols used in “Expected log2(H/M) derivation from known half-lives following protein synthesis inhibition” of Materials and Methods.

t_1/2_	Protein half life
α	Protein synthesis rate
β	Degradation rate constant
τ	Degradation time constant (τ = 1/ β = 1.44· t_1/2_)
*v*	Cell volume
C	Protein concentration
C(∞)	Protein concentration at steady state at Step I (before adding PSI)
C_0_	C(∞) from Step I at Step II (after adding PSI)
α_i_	Protein synthesis rate in the presence of a PSI
k	Fractional protein synthesis rate in the presence of a PSI (k = α_i_/α)

A group of proteins for which we observed statistically significant changes at both 4 and 8 hours, are highlighted at all time points. Interestingly, several of these proteins, in particular Sparc, A2m, Apoe and Sparcl1, were lost at rates that greatly exceed predicted loss rates. Assuming this does not reflect errors in half-life estimates for these proteins, our findings suggest that degradation of these (and probably other) proteins is accelerated when protein synthesis is suppressed.

Examining the detailed time course of protein loss for the proteins highlighted in Figure 10B suggests that protein loss in the presence of CHX occurs rapidly during the first few hours, and then tends to ‘plateau’ (Supplementary Figure S8). In this respect, the findings are reminiscent of the effects of CHX on spontaneous activity levels (Figures 5C,D) raising the possibility that the cells examined here can ‘adapt’ to some extent to the presence of this PSI, perhaps explaining their ability to survive and partially function for several days even in its presence.

**Figure 11:**
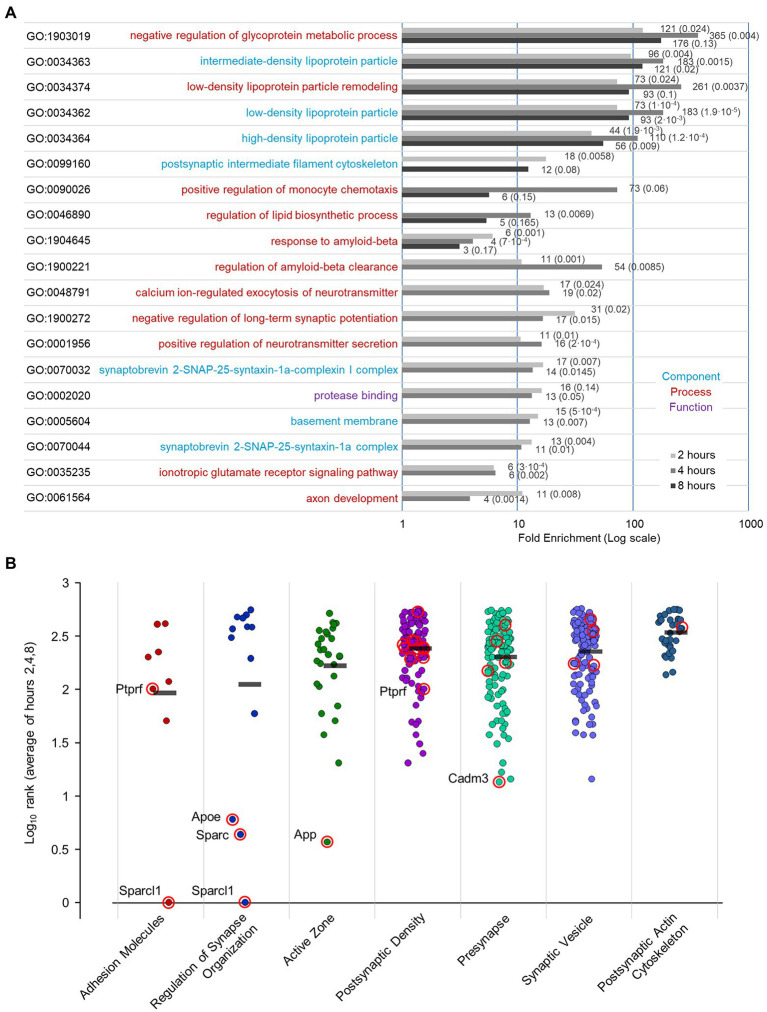
GO term enrichment following PSI treatment, and annotation of proteins with synaptic functions / categories. **(A)** GO enrichment analysis of proteins that were most reduced following PSI exposure. We used GOrilla (Gene Ontology enRIchment anaLysis and visuaLizAtion tool) (Eden et al., 2009; http://cbl-gorilla.cs.technion.ac.il/) to perform the enrichment analysis on filtered lists at each time point (2,4,8 hours) following PSI exposure, ranked with most decreased proteins at the top of list. The top 25% enriched GO terms are displayed along with their fold enrichment 2, 4, and 8 h following exposure to PSIs. Fold enrichment values and FDR values are indicated beside each bar. Terms already enriched at *t* = 0 (probably reflecting statistical artifacts related to the neuronal origin of the samples) were excluded. Further specifics on this bioinformatic analysis are provided in the Materials and Methods section. **(B)** Log_10_ rank (average of hours 2, 4, and 8) of proteins with synaptic functions/categories following protein synthesis suppression. Proteins were ranked at each time point (2, 4, 8 h) such that proteins that decreased the most were at the top of the list. Log_10_ rank average for proteins with the following synaptic functions/categories based on the SynGO knowledgebase (Koopmans et al., 2019; number of proteins in each group from our dataset, and the specific annotations selected for each group are detailed in parentheses below): **Adhesion Molecules** [8 proteins, synapse adhesion between pre- and post-synapse (GO:0099560)], **Regulation of Synapse Organization** [13 proteins, regulation of synapse organization (GO:0050807)], **Active Zone** [29 proteins, presynaptic active zone (GO:0048786), presynaptic active zone cytoplasmic component (GO:0098831), integral component of presynaptic active zone membrane (GO:0099059), structural FIGURE 11 (Continued)constituent of active zone (GO:0098882)], **Postsynaptic Density** [102 proteins, structural constituent of postsynaptic density (GO:0098919), postsynaptic density, intracellular component (GO:0099092), integral component of postsynaptic density membrane (GO:0099061), postsynaptic density (GO:0014069)], **Presynapse** [126 proteins, anchored component of presynaptic membrane (GO:0099026), integral component of presynaptic membrane (GO:0099056), presynapse (GO:0098793), presynaptic cytosol (GO:0099523), presynaptic endocytic zone (GO:0098833), presynaptic endocytic zone membrane (GO:0098835), presynaptic modulation of chemical synaptic transmission (GO:0099171), presynaptic membrane (GO:0042734)], **Synaptic Vesicle** [120 proteins, anchored component of synaptic vesicle membrane (GO:0098993), extrinsic component of synaptic vesicle membrane (GO:0098850), integral component of synaptic vesicle membrane (GO:0030285), regulation of synaptic vesicle cycle (GO:0098693), regulation of synaptic vesicle endocytosis (GO:1900242), regulation of synaptic vesicle exocytosis (GO:2000300), synaptic vesicle (GO:0008021), synaptic vesicle cycle (GO:0099504), synaptic vesicle endocytosis (GO:0048488), synaptic vesicle exocytosis (GO:0016079), synaptic vesicle membrane (GO:0030672), synaptic vesicle priming (GO:0016082), synaptic vesicle proton loading (GO:0097401), synaptic vesicle to endosome fusion (GO:0016189); protein Bassoon was manually excluded from this list], **Postsynaptic Actin Cytoskeleton** [36 proteins, modification of postsynaptic actin cytoskeleton (GO:0098885), postsynaptic actin cytoskeleton (GO:0098871), postsynaptic actin cytoskeleton organization (GO:0098974), regulation of modification of postsynaptic actin cytoskeleton (GO:1905274), structural constituent of postsynaptic actin cytoskeleton (GO:0098973)]. Mean values for each annotated group are marked by a black bar. Proteins with a *p* value ≤0.05 at 4 hours, 8 hours or at both time points (Figure 9), are circled in red.

To further identify protein categories/ functions most affected by PSI exposure we conducted a GO enrichment analysis for the 2, 4, and 8-hour data using GOrilla (Eden et al., 2009).^1^ For this purpose, gene lists were ranked in ascending order (most strongly reduced log_2_H/M ratios at the top), and the significantly enriched GO terms were extracted (Supplementary Table S4; further details are provided in the Materials and Methods section). Interestingly, this analysis revealed, among the top 25% enriched terms, GO terms related to lipid metabolism, amyloid beta clearance, and synapse function (for instance, ionotropic glutamate receptor signaling, synaptobrevin 2-SNAP-25-syntaxin-1a complex; Figure 11A). Finally, we utilized a recently published, expert-curated knowledgebase for the synapse (SynGO; Koopmans et al., 2019) to examine whether synaptic protein groups/annotations were differentially affected by the interruption of protein supply for 2, 4, or 8 hours. To this end, we ranked the proteins at each of these three time points such that proteins that exhibited the greatest reductions were at the top of each list. We then averaged the rank of each protein at the 2, 4, and 8-hour time points for proteins belonging to each of the following synaptic protein groups (Figure 11B; specific terms and protein number for each category are detailed in the Legend): Adhesion Molecules, Regulation of Synapse Organization, Active Zone, Postsynaptic Density, Presynapse, Synaptic Vesicle, and Postsynaptic Actin Cytoskeleton. All synaptic protein groups shown here were downregulated to some degree following PSI exposure, but the effects were not uniform: The greatest effects were observed for “Adhesion Molecules,” and “Regulators of Synapse Organization” (for a complete listing of the proteins in each group, and their Log_10_ average rank, please refer to Supplementary Table S5), which would be in line with the observed effects of acute protein synthesis suppression on synapse counts and synaptic configurations (Figures 3, 6, 7). Interestingly, the latter group includes Sparcl1, recently shown to drive selective increases in excitatory synapse numbers (Gan and Südhof, 2019, 2020).

## Discussion

In this study we conducted experiments designed to reveal aspects of neuronal structure and function that are particularly sensitive to inhibition of protein synthesis. To that end, we abruptly suppressed protein synthesis using the PSIs CHX and ANI, after quantifying their potency to do so at commonly used concentrations. Not unexpectedly, protein synthesis was found to drive cell death, yet at the population level, cell death was protracted, becoming apparent mainly after 48 hours of PSIs exposure. Since we were interested in early events that precede cell death, we focused on an earlier time window of 24 hours or less. Both immunocytochemistry and live imaging indicated that protein synthesis inhibition was associated with some synaptic loss over this time frame, which was more apparent for postsynaptic (compared to presynaptic) reporters. In line with the modest changes measured in counts of presynaptic specializations, evoked synaptic vesicle recycling was not grossly affected by PSIs within this time frame, and effects only became noticeable after longer durations (48 h), with numbers of functional presynaptic sites declining more or less in step with overt cell death. Interestingly, even after 72 hours of PSI exposure and despite widespread cell death, robust synaptic vesicle recycling could still be detected. In contrast, effects on spontaneous network activity became apparent within a few hours of PSI exposure. Similarly, live imaging of fluorescently tagged PSD-95 and Gephyrin (scaffold molecules of excitatory and inhibitory synapses, respectively) revealed reductions in counts of postsynaptic specializations and accelerated rates of synaptic configuration change within the same time frame, more so for excitatory synapses. Reductions in mitochondrial respiration (but not glycolysis) were also observed within this time frame - noticeable after 2 hours and very substantial (approximately 2-fold) after 8 hours.

Given the extent of effects observed within these first hours, we attempted to identify potentially essential proteins lost during this time window. For this purpose, we used dynamic SILAC and Mass Spectrometry to identify proteins lost within 2, 4, and 8 hours of protein synthesis suppression. In general, an excellent association was observed between the propensity of a protein to be lost and its known half-life, although a number of counterexamples raised the possibility that suppressing the provision of newly synthesized proteins might accelerate the degradation of certain proteins. Interestingly, within the group of proteins that exhibited the most significant loss, we found proteins implicated in key cellular functions and pathways, namely, regulators of synapse stability/organization, fatty acid / lipid metabolism, related to cell bioenergetics, and, curiously, related to AD pathology or associated with Amyloid β, a proteolytic cleavage product of APP (amyloid precursor protein) whose fibrillar form is the main component of amyloid plaques found in the brains of AD patients (reviewed in Chen et al., 2017). The predominance of proteins belonging to the first three groups is in general agreement with the structural and physiological effects of PSIs found here. The predominance of the latter group of proteins, however, was unexpected, and may hint that the metabolism of this clinically important group is particularly sensitive to uninterrupted protein synthesis.

### Off-target effects of protein synthesis inhibitors

In this study we used protein synthesis inhibitors to block the supply of new proteins in neurons. Translational inhibitors, however, have been reported to have widespread effects on neuronal function, some of which might be unrelated to their capacity to suppress protein synthesis: Superinduction of immediate early genes (Mahadevan and Edwards, 1991; Hazzalin et al., 1998; Radulovic and Tronson, 2008; Santos et al., 2019); increased synthesis /hyperproduction of specific proteins (Törocsik and Szeberényi, 2000a; Radulovic and Tronson, 2008; Kenney et al., 2016); activation of signaling pathways (Cano et al., 1994; Kardalinou et al., 1994; Zinck et al., 1995; Iordanov et al., 1997; Hazzalin et al., 1998; Iordanov and Magun, 1998; Törocsik and Szeberényi, 2000a; Monaghan et al., 2014; Tyssowski et al., 2018); apoptosis/cytotoxicity (in certain cell types; Törocsik and Szeberényi, 2000b; Monaghan et al., 2014; Chan et al., 2017); effects on protein degradation (Franklin and Johnson, 1998; Dai et al., 2013; see also Ding et al., 2007; Kaang and Choi, 2012; Jarome and Helmstetter, 2014); and altered axonal transport (Levy et al., 1990). Despite these reports, the similarity of the findings in experiments based on two different inhibitors (CHX, ANI) suggest that these are unlikely to stem primarily from off-target effects of these inhibitors.

### Limitations of proteomics-based methods

Proteome-wide studies are biased towards abundant proteins, and thus, it is almost certain that our proteomic analyses failed to detect many proteins lost rapidly upon PSI exposure. Thus, some proteins with known short half-lives (e.g., Munc13; UNC13A) did not show up as early-lost proteins. Even within the population of identified proteins, relatively few passed the threshold for inclusion in the group of proteins that were considered to change significantly, in spite of the excellent fit between measured protein levels and predictions based on known half-lives for hundreds of additional proteins (Figure 10B). What these are, and how they might explain the structural and functional effects described above (as well as others not explored here) remains to be determined. Perhaps new methods that also provide the spatial (cellular/subcellular) resolution that proteome-wide studies lack will gradually elucidate their identity and importance (e.g., Elamri et al., 2018; Truckenbrodt et al., 2018; Heumüller et al., 2019; Biever et al., 2020; Sun et al., 2021).

### Implications for synaptic maintenance

The provision of newly synthesized proteins is essential for maintaining the organization and function of both presynaptic and postsynaptic specializations; our data indicate, however, that the presynaptic compartment might be somewhat more resilient in this regard. For example, during the first 24 hours of PSI exposure, reductions in counts of presynaptic specializations based on a presynaptic reporter (Synapsin; Figure 3A) were was not as apparent as those observed with postsynaptic reporters (PSD-95, Gephyrin; Figures 3B, 6B,E). Similarly, changes in vesicle recycling capacity occurred relatively slowly, with reductions in functional presynaptic bouton number roughly tracking rates of cell death (Figure 4). This apparent resilience would seem to be in accordance with the extraordinarily long supply lines of presynaptic replenishment (e.g., Alvarez et al., 2000; Rosenberg et al., 2014; Ziv, 2018; Aiken and Holzbaur, 2021; see Introduction). Conversely, the preservation of presynaptic function seems at odds with the short half-lifetimes reported for key presynaptic proteins such as the active zone proteins Munc13 and RIM (Südhof, 2012; reviewed in Ziv, 2018; Cohen and Ziv, 2019).

In contrast, notable changes to postsynaptic organization were apparent within hours of PSI exposure (Figures 6B,E). These manifested as reductions in counts of PSD-95:EGFP and mCherry:Gephyrin puncta, reductions in PSD-95:EGFP fluorescence (Figure 6C; Supplementary Figures S4A,B), and the destabilization of synaptic configurations (Figure 7). These effects are unlikely to be secondary to reductions in network activity (Figure 5), as such reductions have been shown to drive synaptic *growth*, and *reduce* synaptic configuration change rates (Minerbi et al., 2009; Hazan and Ziv, 2020). It is interesting to speculate that PSI-induced synaptic loss reflects diminished stabilization of newly formed, small synapses (Hazan and Ziv, 2020; Kasai et al., 2021) due to the loss of Sparcl1 (Gan and Südhof, 2020) and its likes. Indeed, “Adhesion Molecules” and “Regulators of Synapse Organization” were among the synaptic groups in which proteins were lost to the greatest extent following 8-hour PSI exposures.

We observed that PSI exposure led to rapid declines in spontaneous network activity; in similar experimental conditions, mitochondrial respiration was significantly reduced within similar time frames, hinting that reductions in spontaneous network activity levels stem from membrane depolarization. Indeed, the maintenance of Na^+^ and K^+^ ionic gradients depends on ATP-driven Na^+^/K^+^ pumps, which are major neuronal ATP consumers (reviewed in Attwell and Laughlin, 2001; Engl and Attwell, 2015; Lezmy et al., 2021). In this respect, synaptic vesicle-resident V-ATPases, which create the proton gradients that drive the concentration of neurotransmitter within vesicles, have been shown to represent another major source of energy consumption (Pulido and Ryan, 2021; for a recent comprehensive review on presynaptic energy homeostasis, see Li and Sheng, 2022). We mention this because we cannot exclude the possibility that synaptic vesicle recycling observed in the presence of PSIs was in fact dysfunctional due to impaired vesicle filling, entailing further reductions in spontaneous activity levels.

### Relevance to neurodegeneration

Unexpectedly, proteins related to AD pathology or associated with Aβ (Apoe, App, Itm2c, Cst3, C1qb, Sparc, A2m) were among the proteins most strongly affected by PSI exposure, hinting that this group might be especially sensitive to uninterrupted protein synthesis. Apoe, one of the proteins in this group, has mechanistic connections to several of the effects we observed here. First, Apoe is a major regulator of lipid homeostasis (e.g., Lanfranco et al., 2020), serving as a key player in fatty acid (FA) metabolism, by shuttling FA from neurons to astrocyte mitochondria where FAs are processed to produce ATP; the ATP produced in astrocytes then activates interneurons, driving increases in inhibitory transmission (Bowser and Khakh, 2004; Ioannou et al., 2019; Qi et al., 2021). Second, Apoe is directly involved in synapse elimination (Singhrao et al., 1999; Stevens et al., 2007; Schafer et al., 2012; Sekar et al., 2016; Dejanovic et al., 2018; Kang et al., 2018; Metaxas et al., 2019; Vukojicic et al., 2019; Wu et al., 2019; Yin et al., 2019; Datta et al., 2020; Györffy et al., 2020; Savage et al., 2020; Werneburg et al., 2020). Synapse elimination almost certainly drives forgetting, linking the effects observed here with classic (amnestic) PSI effects (Wang et al., 2020; for reviews see Davis and Squire, 1984; Hernandez and Abel, 2008; Kasai et al., 2021). Along these lines, accelerated memory loss in older human individuals at risk for AD was found to be associated with a single nucleotide polymorphism in Sparcl1, which was also associated with reduced Sparcl1 expression (Seddighi et al., 2018b). As mentioned above, Sparcl1, one of the most strongly affected proteins identified in our study, plays important roles in synapse formation (Gan and Südhof, 2020) and possibly synapse stabilization.

App, the second protein in the aforementioned list, has also been shown to regulate synaptic maintenance or drive synaptic loss. Two recent studies are particularly relevant in this regard: The first study (Bold et al., 2022) showed that APPsα (the proteolytic cleavage product of APP along the nonamyloidogenic pathway, i.e., the one not producing Aβ) rescued abnormally low spine density in tauopathy model mice, even when applied at nanomolar concentrations. In contrast, the second study (Lee et al., 2022) as well as many others, showed that soluble forms of oligomeric Aβ (the proteolytic APP cleavage product along the amyloidogenic pathway) drove excitatory synapse loss. Intriguingly, this study provided compelling evidence for causal links between Aβ-triggered loss of postsynaptic specializations (comparable both in extent and time course to Figure 6 above) and an upstream loss of dendritic mitochondria biomass, and by extension, compromised energy production capacity. These findings may suggest that many of the PSI-related physiological and molecular changes we reported here converge on faulty synaptic maintenance.

Synapse elimination is a hallmark of neurodegeneration; in some disorders it results directly from reduced translation capacity, which has been postulated to be among the underlying causes of multiple disorders including Down Syndrome, Rett Syndrome, Alzheimer’s Disease, Parkinson’s Disease, and neuronal degeneration in prion-diseased mice (Kelleher and Bear, 2008; Darnell and Klann, 2013; Buffington et al., 2014; Ramiro-Cortés et al., 2014; Wu et al., 2016; MacDonald et al., 2017; Costa and Willis, 2018; Roeper, 2018; Soukup et al., 2018; Xu et al., 2018; Bastin et al., 2019; Blumenstock et al., 2019; Cai et al., 2019; Jackson et al., 2019; Osimo et al., 2019; Henstridge et al., 2019a,b; Cefaliello et al., 2020; Licznerski et al., 2020; Lo and Lai, 2020; Smith et al., 2020). Prime examples are neurodegenerative diseases associated with the buildup or aggregation of misfolded proteins (prion disease and others, including Alzheimer’s disease and Parkinson’s disease) linked to defective protein quality control. These conditions have been shown to result in the shutdown of global protein synthesis through the unfolded protein response and others, such as the integrated stress response (reviewed in Mallucci et al., 2020; Sidhom et al., 2022). Moreover, drugs aimed at reversing this shutdown have shown clinical promise, suggesting that biochemical mechanisms and key players driving translation downregulation in such conditions might constitute targets for combating neurodegenerative disease (reviewed in Gonzalez-Teuber et al., 2019; Hughes and Mallucci, 2019; see also Charif et al., 2022). Our observation that synapse elimination precedes overt cell death is in line with these findings and the view that synapses are lost early, preceding or independent of neuronal death (Dejanovic et al., 2018; Soukup et al., 2018) albeit on a greatly compressed time scale, possibly reflecting the severity of the experimental manipulations used here.

### Concluding remarks

The findings described here point to neuronal excitability, energy supply and stability of postsynaptic specializations as early-occurring failure points under conditions of compromised supply of newly synthesized protein copies. We mention in closing that PSIs have a long history of use in studies aiming to uncover the importance of new protein synthesis for memory consolidation on time scales of a few hours (Rosenberg et al., 2014). The failure points observed here and their comparable timelines would seem to prompt some care in interpreting such experiments, in accordance with previous suggestions along these lines (e.g., Sharma et al., 2012; Greenberg et al., 2014).

## Materials and methods

### Animal welfare

Experiments were performed in primary cultures of newborn rat neurons prepared according to a protocol approved by the “Technion, Israel Institute of Technology Committee for the Supervision of Animal Experiments.”

### Cell culture

Primary cultures of cortical neurons for live imaging experiments and immunocytochemistry were prepared as described previously (Tsuriel et al., 2006; Minerbi et al., 2009). In brief, cortices of 1–2 days-old rats of either sex (Wistar, Charles River, United Kingdom) were dissected, dissociated by trypsin treatment followed by trituration using a siliconized Pasteur pipette and plated onto 22 × 22 mm coverslips coated with polyethylenimine (Sigma) inside 8-mm-diameter glass cylinder microwells (Bellco Glass). For live imaging and electrophysiology recording experiments, cells were plated on MEA dishes as in Minerbi et al. (2009). Cells were initially grown in medium containing Minimum Essential Medium (MEM; Sigma), 25 mg/l insulin (Sigma), 20 mM glucose (Sigma), 2 mM L-glutamine (Sigma), 11 mg/l gentamycin sulfate (Sigma), and 10% NuSerum (Becton Dickinson Labware). The preparations were then transferred to a humidified tissue culture incubator and maintained at 37°C in a 95% air and 5% CO_2_ mixture. Half the volume of the culture medium was replaced every 7 days with fresh feeding medium similar to the medium described above but devoid of NuSerum, containing a lower concentration of L-glutamine (Sigma, 0.5 mM), and 2% B-27 supplement (Gibco).

Primary cultures of rat cortical neurons used for SILAC experiments were prepared as described above. 10^6^ or 1.2·10^6^ cells were then plated in 12-well plates whose surface had been pretreated with polyethylenimine (Sigma) to facilitate cell adherence and then maintained at 37°C in a 95% air and 5% CO_2_ humidified incubator. Half the volume of the culture medium was replaced three times a week with fresh feeding medium as described below (‘*Dynamic SILAC*’).

### Protein synthesis inhibition experiments using HaloTag fusion protein

HaloTag-mTurq2 was generated as described previously (Cohen et al., 2020) as was the non-fluorescent HaloTag blocker CPXH [1-chloro-6-(2-propoxyethoxy)hexane]. Halo-Tag mTurq2 was expressed by calcium phosphate transfection as described previously (Bresler et al., 2004), or by adding HaloTag-mTurq2 lentiviral particles to the neuronal cultures (as detailed below). Experiments were carried out on neurons grown in culture for 2.5 to 3 weeks. The procedure detailed in Cohen et al. (2020) was followed: The original cell culture media was set aside and the CPXH was applied for 30 min (10 μM in cell culture media) to block free HaloTag binding sites. The cells were then washed three times in cell culture media and the original media was returned. Cycloheximide (100 μg/ml, Sigma), anisomycin (25 μM, Sigma), puromycin (1 μM, Sigma), puromycin aminonucleoside (1 μM, Sigma), or carrier solution (water or DMSO) were then applied and the cells were returned to a 37°C, 5% CO_2_ incubator. Twenty-four hours later, the HaloTag ligand Janelia Fluor 635 (JF635HT; Grimm et al., 2017; 100 nM) was added to the cells for 30 min, and the cells were imaged immediately.

### Immunolabeling against synaptic proteins following protein synthesis inhibition

At days 15–19 in culture (2.5 weeks after plating), primary cultures of cortical neurons prepared as described above were treated with protein synthesis inhibitors: anisomycin, cycloheximide, or puromycin dihydrochloride (all purchased from Sigma) at a final concentration of 25 μM, 100 μg/ml (~355 μM), or 1 μM, respectively, or carrier solution (either water, or DMSO at 0.1% final concentration). The cells were fixed after 24 h, and stained using antibodies for synaptic proteins. Immunolabeling was performed by washing the cells in Tyrode’s solution (119 mM NaCl, 2.5 mM KCl, 2 mM CaCl_2_, 2 mM MgCl_2_, 25 mM HEPES, 30 mM glucose, buffered to pH 7.4) followed by fixation with 4% paraformaldehyde (PFA) and 120 mM sucrose in PBS (fixative solution) for 20 min, or cold (−18°C) methanol for 5 min. PFA-fixed cells were permeabilized for 10 min in fixative solution to which 0.25% Triton X-100 (Sigma) was added. The cells were washed three times in PBS, incubated in 10% bovine serum albumin (BSA, Sigma) for 1 h at 37°C, and incubated overnight at 4°C with primary antibodies in PBS and 1% BSA. The cells were then washed three times for 5 min with PBS and incubated for 1 h at room temperature with secondary antibodies in PBS and 1% BSA. The cells were washed again with PBS three times, and imaged immediately.

Primary antibodies included: mouse anti-synapsin I 1:400 (Transduction Laboratories); mouse anti-PSD-95 1:350 (Clone K28/43, Upstate Biotechnology); Secondary antibodies (used at a dilution of 1:200) included: AlexaFluor 633 nm goat anti-mouse IgG2a (Invitrogen), Cy5 donkey anti-mouse (Jackson Immuno Research Laboratories).

### Cell viability testing

Propidium Iodide and Calcein-AM (Sigma) were applied as in Sadeh et al., 2015 and Sadeh et al., 2016, to assess neuronal viability following protein synthesis inhibitor treatment. Age-matched pairs of neuronal cultures, 2 to 2.5 weeks after plating, were treated with protein synthesis inhibitor (CHX, 100 μg/ml, Sigma or ANI, 25 μM, Sigma, or carrier solution at matching volumes) for 24, 48, 72, or 96 hours. During protein synthesis inhibitor treatment, the neurons were maintained in a humidified, 37°C, 5% CO_2_ incubator. At the end of these periods, Calcein-AM (final concentration 2 μM) was added to the cell culture media for 30 min. Propidium Iodide (final concentration 2.5 μM) was added to the cell culture media just before imaging (for less than 10 min). Twenty fields of view per cylinder were imaged, two cylinders per time point and condition. Three separate experiments (separate cell culture preparations from different rat pup litters, PSI/control treatments, labelling and analysis) were carried out in this fashion with CHX/DMSO and two with ANI/Water. Live cell counts per field of view (Calcein-positive cells) and dead cell counts per field of view (Propidium Iodide-positive cells) were obtained using ImageJ software. Cell viability in each cylinder was assayed by calculating the average ratio (from all fields of view) of live cell count to total (live + dead) cell count for each duration.

### FM loading/destaining

FM4-64 labeling of functional presynaptic boutons was done by flooding the perfusion chamber with Tyrode’s solution (formulation detailed above) containing the functional endocytosis marker FM4-64 (N-(3-Triethylammoniumpropyl)-4-(6-(4-(Diethylamino) Phenyl) Hexatrienyl) Pyridinium Dibromide, 15 μM; Invitrogen). Dye loading was done by field stimulation of the neurons at 20 Hz for 60 s, followed by a 30 s pause, and then vigorous washes with Tyrode’s solution for 60 s. Dye unloading (destaining) was done by field stimulation at 20 Hz for 120 s. The Tyrode’s solution contained glutamate receptor antagonists: 6-cyano-7-nitroquinoxaline-2,3-dione (CNQX, 10 μM; Sigma) or 2,3-dioxo-6-nitro-1,2,3,4-tetrahydrobenzo[*f*]quinoxaline-7-sulfonamide (NBQX, 10 μM; Tocris), and D,L-2-amino-5-phosphonopentanoic acid (APV, 50 μM; Sigma) to minimize network reverberations. The experiments were performed at 36°C, 14–16 days after plating.

### Lentivirus production and transduction

The construct used to produce HaloTag-mTurq2 lentiviral particles was generated as described previously (Cohen et al., 2020). The construct used to express PSD95:EGFP was described previously (Minerbi et al., 2009). The construct used to express mCherry:gephyrin was generated from mTurq2-Gephyrin (Rubinski and Ziv, 2015) as follows: The mCherry sequence from cloning vector pAW8_mCherry (complete sequence GenBank: EU855181.1) was used for *de novo* synthesis of an AgeI-mCherry-BsrGI segment, that was inserted between AgeI and BsrGI sites in mTurq2-Gephyrin instead of mTurq2. Cloning and gene synthesis were done by Genscript (Piscataway, NJ, USA).

Lentiviral particles were produced in house using a commercial kit (ViraPower™, ThermoFisher Scientific). Briefly, 80%-confluent HEK293T cells were transfected using Lipofectamine 2000 (Invitrogen) with a mixture of three Virapower kit packaging plasmids (pLP1, pLP2, pLP/VSVG; packaging vector mix of the ViraPower four-plasmid lentiviral expression system), and the expression vector. Lentiviral stocks were prepared by collecting the supernatant 48 hours after transfection, filtering it by a 0.45-μm filter, and storing the liquid in small aliquots at −80°C. Neuronal transduction was carried out 5 days after plating by dropwise addition of 10–20 μl of concentrated virus (optimized for each viral stock) to each cortical neuronal culture. For experiments with HaloTag-mTurq2, the DNA was introduced in cultures of neuronal cells growing in 8-mm cloning cylinders, 4 days after plating the neurons, by adding 1.5 μl of concentrated virus to each cortical neuronal culture.

### Microscopy and image analysis

Imaging of immunolabeled neurons in protein synthesis inhibition experiments was performed using a custom-built confocal laser scanning microscope (Tsuriel et al., 2006) using a 40X, 1.3 NA Plan-Fluar objective. Excitation was performed at 633 nm (Helium Neon laser). Fluorescence emissions were read using a 635 nm (Chroma) or a 650 nm long-pass filter (Semrock). Images were collected by averaging six frames at two to four focal planes spaced 0.8 μm apart. All data were collected at a resolution of 640 × 480 pixels, at 12 bits per pixel. Image analysis was performed using custom written software (“OpenView”) written by NEZ. Analysis was performed on maximal intensity projections of 2 sections, located 0.8 μm apart. Intensities of fluorescent puncta were measured by programatically centering 9 × 9 pixel (~1.4 μm × 1.4 μm) regions of interest on individual puncta and obtaining the average fluorescence intensity for each area. Programmatic puncta detection was based on identifying local fluorescence maxima in radii of 5 pixels, after preprocessing images with a Mexican hat filter.

For FM4-64 staining/destaining experiments, excitation was performed using a 488 nm laser. Emissions were read through a 635 nm long pass filter (Chroma). Intensity values for individual puncta were extracted from OpenView by centering 10 × 10 pixel boxes over puncta, and expressed as a percentage of initial fluorescence intensity of each punctum before destaining. Puncta with mean intensity values in the baseline window (i.e., the first 10 imaging cycles, prior to electrical stimulation) lower than 600 AUs were excluded. The remaining values were then pooled, averaged and plotted per condition in Microsoft Excel.

Imaging of neurons for live-dead assays after protein synthesis inhibitor treatment was performed by averaging six frames at a single Z-section. Neurons were imaged in a 5% CO_2_ chamber, with the microscope objective heated to 36°C. Excitation of Calcein-AM was performed at 488 nm. Emissions were read through a 500–550 nm band pass filter (Chroma). Excitation of Propidium Iodide was performed at 514 nm. Emission was read using a 635 nm long-pass filter (Chroma). Data analysis was performed using ImageJ software; data were thereafter exported to Microsoft Excel for further analysis.

For live imaging experiments with HaloTag-mTurquoise2 and HaloTag ligands, mTurq2 excitation was performed at 457 nm (Argon laser). Emission was read using either 467–493 nm or 465–485 band pass filters. JF635HT excitation was performed at 633 nm (Helium Neon laser). Emissions were read using a 635 nm long-pass filter (Semrock). Images were collected by averaging six frames at three focal planes spaced 0.8 μm apart. All data were collected at a resolution of 640 × 480 pixels, at 12 bits per pixel. Image analysis was performed using OpenView by placing rectangular regions of interest on cell bodies of mTurq2-positive neurons using the mTurq channel. Fluorescence values of all channels were then collected from these regions and average values were calculated for each neuron. To correct for background fluorescence and non-specific labeling, regions of interest were placed on mTurq2-negative somata, and these values were subtracted from all fluorescence readings for all channels.

Long-term imaging experiments in MEA dishes were done as follows: Neurons were grown on thin-glass MEA dishes (180 μm thickness; Minerbi et al., 2009), which allow for high-resolution imaging using high numerical aperture, oil immersion objectives. Time-lapse recordings were performed by averaging six frames at each of 10 focal planes spaced 0.8 μm apart. Excitation of PSD95:EGFP was performed at 488 nm. Excitation of mCherry:gephyrin was performed at 594 nm. Fluorescence emissions were read using 500–550 nm band pass (Chroma) and 594 nm long pass filters, respectively. All data were collected at a resolution of 640 × 480 pixels, at 12 bit/pixel with the confocal aperture fully open. Data were typically collected from 12–18 sites in each dish using the microscope robotic stage to cycle automatically through all the selected sites at 1-hour time intervals. Focal drift was corrected automatically using the microscope autofocus feature.

Images were analyzed by centering rectangular areas of 7 × 7 pixels (~1 × 1 μm) on synaptic puncta, and mean pixel intensities within the areas were obtained from maximal intensity projections of Z-section stacks (ten images at focal planes spaced 0.8 μm apart). Puncta whose fluorescence intensities approached saturation were disregarded.

To track individual identified puncta, areas were initially placed over all puncta and a smaller subset (typically 50–90 per site) was thereafter tracked. All tracking was first performed automatically by OpenView, and subsequently verified and corrected manually where deemed necessary. Puncta for which tracking was ambiguous were discarded and only puncta tracked successfully for the entire time course of the experiment were included in the analysis. To minimize the effects of short term fluctuations and measurement noise, measurements for individual puncta were first smoothed using a 3-time point (3-hour) low pass filter and these smoothed data were used for all further analysis. Because expression levels (for PSD-95:EGFP and mCherry:Geph) varied slightly from one neuron to another, puncta fluorescence data from each neuron were first normalized to mean puncta fluorescence of that neuron (this was done separately for each of the expressed fusion proteins), allowing for pooling of data from different neurons and experiments, correcting for differences in expression levels between experiments. Thus, fluorescence intensity levels are expressed as fractions of the mean fluorescence (per each fusion protein) at initial time points.

To measure distributions of puncta intensities, areas were placed programatically on fluorescent puncta at each time step using identical parameters, but without tracking individual puncta.

### Experimental conditions in long-term experiments

MEA dishes were covered by a custom designed cap containing inlet and outlet ports for perfusion media and CO_2_/air mixture, a submerged circular platinum wire serving as a reference (ground) electrode, and a small removable glass cap to allow for sterile application of pharmacological agents to the MEA dish during the experiment. The MEA dish was perfused with warm feeding media throughout the entire experiment (for exact formulation see “*Cell Culture*” section above), starting at 24 hours prior to the imaging and electrical recordings, as a “pre-baseline” to allow the network to settle and reach a steady state on the setup before starting the recordings. The dish was perfused at a constant, ultra-slow rate of 2.5 ml/day by a custom-built perfusion system based on a peristaltic pump (Instech laboratories, United States) and silicon tubing. The dish was streamed at very slow rates with a sterile-filtered mixture of 5% CO_2_ and 95% air through the CO_2_ inlet in the cap. Flow rates were regulated by a high-precision flow meter (Gilmont Instruments, United States). The base of the headstage and the microscope objective were heated to 37°C and 36°C, respectively, using separate temperature controllers, resulting in a controlled temperature of 36–37°C in the media within the dish.

### Recordings of network activity

Cortical neurons were plated on thin glass multielectrode array (MEA) dishes at densities identical to those used for SILAC experiments (see above). MEA dishes used here contained 59, 30 μm diameter, electrodes arranged in an 8 × 8 array, spaced 200 μm apart (Multichannel Systems). The dishes were covered by a custom-designed cap containing a submerged platinum wire loop serving as a ground electrode, heated to 37°C, and provided with a filtered stream of 5% CO_2_ and 95% air through an inlet in the cap. Network activity was recorded through a commercial 60-channel headstage/amplifier (Inverted MEA1060, MCS) with a gain of 1,024x and frequency limits of 1-5,000 Hz. Data acquisition was performed using AlphaMap (Alpha-Omega) or custom software – Closed Loop Experiment Manager (CLEM; Hazan and Ziv, 2017). All data were stored as threshold crossing events, with the threshold set to -30 μV. Electrophysiological data were imported to Matlab (MathWorks, MA, USA) and analyzed using custom written scripts. The effects of protein synthesis inhibitors on neuronal network activity were measured by adding anisomycin (25 μM, Sigma) or cycloheximide (100 μM or 100 μg/ml; ~355 μM, Sigma) to the MEA dishes after 5 hours of baseline recording (values denote the final concentrations in the dish and in the perfusion media). Recordings were then continued for another 20 hours. For long-term experiments, inhibitors were added following at least 24 h of pre-baseline perfusion with warm cell culture media and an additional 24 h of baseline recordings, followed by recordings for at least another 24 h. In these experiments, inhibitors were simultaneously added to the perfusion media to maintain the desired inhibitors concentrations throughout the experiments.

### Bioenergetic profiles

Rat cortical neurons prepared as described above were plated onto Seahorse XF 96-Cell Culture Microplates (Agilent) whose surface had been pretreated with polyethylenimine (Sigma) to facilitate cell adherence, at a density of 50,000 cells/ml (150 μl seeded in each microwell). Cultures were maintained at 37°C in a 95% air and 5% CO_2_ humidified incubator, replacing half of the volumes with fresh medium every 2–3 days until the day of the assay (18–21 days after plating).

The day before the assay, the sensor cartridge was hydrated overnight in a non-CO_2_, 37°C enclosure, by adding 200 μl of sterile water to each well of the utility plate. On the day of the assay the water was replaced with 200 μl of pre-warmed Seahorse XF calibrant solution (Agilent) and incubated for 1 hour at 37°C in a non-CO_2_ enclosure. Compounds for the Mito Stress Test and glycolysis assay (see below) were then added to the reagent ports and the cartridge was placed in the Agilent Seahorse XFe96 Analyzer for calibration.

On the day of the assay, protein synthesis inhibitor Cycloheximide (100 μg/ml, Sigma) or vehicle (DMSO at matching volume) was added to the cells for 2 or 8 hours. Two hours before the assay, the cells were washed three times with assay media: DMEM media without glucose, L-glutamine, phenol red, sodium pyruvate, and sodium bicarbonate (D5030; Sigma), adjusted to pH 7.4 to which pyruvate, glutamine, and glucose were added to a final concentration of 1 mM, 2 mM, and 10 mM, respectively. The microwell volume at the beginning of the assay was 180 μl. cycloheximide (or DMSO) were also added during the washes. The cell plate was then incubated for ~1.5 h in a non-CO_2_ incubator at 37°C to allow CO_2_ diffusion from the cells, medium, and plate. The cells were observed under a microscope before and after all washes and incubation steps to ensure cell uniformity and integrity, and ascertain the good general condition of the cells.

After the non-CO_2_ incubation, the cell plate was placed in the Agilent Seahorse XFe96 Analyzer for measuring oxygen consumption rate (OCR) and extracellular acidification rate (ECAR). The protocol, essentially a Mito Stress Test with added glycolytic measurements, consisted of basal respiration measurements, followed by sequential administration of the following compounds: oligomycin (2 μM), carbonyl cyanide 4-(trifluoromethoxy) phenylhydrazone (FCCP; 2 μM), and rotenone/antimycin A (Rot/AA; 2 μM in 2-h experiments, 4 μM in 8-h experiments), and 2-deoxyglucose (2-DG; 50 mM). All compounds were prepared in fresh assay media immediately before use.

Five cycles of measurements were performed in the baseline measurement and after each compound addition, and OCR and ECAR values were recorded. Each measurement was acquired over a 3-min period, followed by a one-minute mixing period instead of the typical 3 minutes to reduce mechanical disturbance to the thin neuron layer while still allowing for concentration equilibration after each measurement and probe lifting. The protocol allowed estimation of basal mitochondrial respiration, proton leak, spare capacity, maximal respiration, and residual (non-mitochondrial) respiration.

Data acquisition and analysis were performed using Seahorse XFe96 Wave software (Agilent Technologies, v 2.6.0.31). The data were subsequently exported to Microsoft Excel for further analysis. Values measured from each well were normalized to background levels (the wells on the plate perimeter, in which no cells were seeded, and which contained only assay media), and to the fifth baseline measurement, which is assumed to represent the most stable value. To allow comparison across wells and cell plates, values were expressed as the percent change from baseline values. Samples that failed to respond to FCCP or Rot/AA, or displayed *increased* OCR values following oligomycin administration (reflecting pharmacological agent delivery failures), were excluded from the analyses of respiration measures.

### Dynamic SILAC

Cortical cells were grown for 14 days in lysine and arginine-free MEM (custom-made MEM-Eagle Earle’s Salts Base, without lysine and arginine; Biological Industries) to which “heavy” (H) variants (Lys8-^13^C_6_, ^15^N_2_; Arg10-^13^C_6_, ^15^N_4_) or “medium” (M) variants (Lys6-^13^C_6_; Arg6-^13^C_6_; Cambridge Isotope Laboratories) were added so that final concentrations (0.4 mM and 0.6 mM respectively) were identical to the nominal lysine and arginine concentrations in standard cell culture media. After 2 weeks in culture, protein synthesis inhibitor or vehicle (DMSO/water) was added to the cells. The cells remained in the “heavy” or “medium” cell culture media to which the inhibitors were added directly. Harvesting was done either immediately after adding the inhibitor, or after 2, 4, and 8 hours. The neurons were harvested by gently washing the cells 3 times in a physiological solution (“Tyrode’s,” formulation as detailed above), aspirating all the solution, and immediately adding 100 μl of lysis buffer composed of 10% Sodium Dodecyl Sulfate (SDS; Sigma), 30 mM TRIS HCl (Sigma), 3.4% glycerol, 25 mM DTT (Sigma), and 0.5% v/v protease inhibitor (Calbiochem). The cells were then scraped in the lysis buffer using a disposable cell scraper. The lysate was then collected, pipetted vigorously on ice, boiled for 5 min, and frozen at −80°C until used. The samples were mixed together as pairs of time-matched treatment and control samples.

### In gel proteolysis and mass spectrometry analysis

Thirty micrograms of protein from each time point were separated on 4–15% SDS-PAGE (polyacrylamide gel electrophoresis), and each gel lane was sliced into five sections. The proteins in each gel slice were reduced with 3 mM DTT (60°C for 30 min), modified with 10 mM iodoacetamide in 100 mM ammonium bicarbonate (in the dark, room temperature for 30 min) and digested in 10% acetonitrile and 10 mM ammonium bicarbonate with modified trypsin (Promega) overnight at 37°C, at a 1:10 enzyme-to-substrate ratio. An additional second trypsinization was done for 4 hours.

The tryptic peptides were desalted using C18 tips (Top tip, Glygen) dried and re-suspended in 0.1% Formic acid.

They were analyzed by LC–MS/MS using a Q Exactive HF mass spectrometer (Thermo) fitted with a capillary HPLC (easy nLC 1,200, Thermo). The peptides were loaded on a homemade capillary column (30 cm, 75 μm internal diameter) packed with Reprosil C18-Aqua reversed phase material (Dr Maisch GmbH, Germany) in solvent A (0.1% formic acid in water). The peptide mixture was resolved with a 5–28% linear gradient of solvent B (95% acetonitrile, 0.1% formic acid) for 120 min followed by a 15-min gradient of 28–95% and 25 min at 95% acetonitrile with 0.1% formic acid in water at a 0.15 μl/min flow rate.

Mass spectrometry was performed in a positive mode (m/z 300–1800, resolution 120,000 for MS1 and 15,000 for MS2) using repetitively full MS scan followed by collision induces dissociation (HCD, at 27 normalized collision energy) of the 18 most dominant ions (>1 charges) selected from the first MS scan. The AGC settings were 3 × 106 for the full MS and 1 × 105 for the MS/MS scans. The intensity threshold for triggering MS/MS analysis was 1 × 104. A dynamic exclusion list was enabled with exclusion duration of 20 s.

The mass spectrometry data was analyzed using the MaxQuant software 1.5.2.8 (Cox and Mann, 2008) for peak picking and identification using the Andromeda search engine, searching against the rat proteome from the Uniprot database with mass tolerance of 6 ppm for the precursor masses and 20 ppm for the fragment ions. Oxidation on methionine, phosphorylation on STY, gly-gly on K, and protein N-terminus acetylation were accepted as variable modifications, and carbamidomethyl on cysteine was accepted as static modification. Minimal peptide length was set to six amino acids and a maximum of two miscleavages was allowed. Peptide- and protein-level false discovery rates (FDRs) were filtered to 1% using the target-decoy strategy. Protein tables were filtered to eliminate identifications from the reverse database, common contaminants, and single peptide identifications. The data were quantified by SILAC analysis using the same software. H/L, M/L, and H/M ratios for all peptides belonging to a particular protein species were pooled, providing a ratio for each protein.

### Data analysis of SILAC experiments

For SILAC experiments with protein synthesis inhibitors, a total of five experiments using Cycloheximide (each with four time points: 0, 2, 4, and 8 hours) were performed. As described above, SILAC data samples were mixed together as pairs of time-matched experiment and control sets, and run together on preparative gels. To correct for potential remaining differences in the amounts of material collected from the experiment and control samples, we normalized all H/M ratios to the H/M ratios measured for a group of abundant proteins, previously shown to be exceptionally long-lived (Toyama et al., 2013; Table 1). H/M ratios were normalized separately for each experiment and time point, and displayed as log_2_(H/M). Analyses were performed on data from proteins for which H/M ratios were measured for at least five peptides in total, from at least three experiments. Statistical testing was performed by two-sided t-test for the biological repeats (each time point compared to 0 hours, with a cutoff value of *p* ≤ 0. 05 considered statistically significant).

### Expected log_2_(H/M) derivation from known half-lives following protein synthesis inhibition

The derivation is broken into two steps – before and after PSI addition (A summary of the meaning of each symbol is provided in Table 2).

**Step I:** Before adding the protein synthesis inhibitor.

Let us assume that **(i)** At some time = *t*, a cell contains some amount of a particular protein; **(ii)** The synthesis of this protein occurs as a zero order reaction denoted as αv , indicating the mass per volume (concentration) synthesized per unit time; **(iii)** The degradation rate occurs as a first order reaction where the rate constant is β=1/τ , τ being the time constant of the degradation reaction such that τ = 1.44 t_½_ (t_½_ is the proteins half-life time).

The change in protein concentration *C* as a function of time depends on its synthesis and degradation rates, and is given by the expression:


$$\frac{dC}{dt}=\frac{\alpha }{v}-\beta C\left(t\right)$$

Assuming the protein concentration at ( t→∞) is at steady state:


$$\frac{dC}{dt}=0_{\left(t\to \infty \right)}$$

$$\frac{\alpha }{v}=\beta C_{\left(\infty \right)}$$

$$\frac{dC}{dt}=\beta C_{\left(\infty \right)}-\beta C\left(t\right)$$

And denoting $C^{\prime }=\frac{C\left(t\right)}{C_{\left(\infty \right)}}$ :


$$\frac{dC^{\prime }}{dt}=\beta -\beta C^{\prime }$$

Solving for C′ with the condition that the initial concentration $C^{\prime }\left(0\right)=0$ results in:


$$C^{\prime }=1-e^{-\beta t}$$

**Step II:** The system has reached steady state. Now, $C_{0}$ will be the $C_{\left(\infty \right)}$ of Step I.

Now, a protein synthesis inhibitor is added. The rate of protein synthesis is slowed by the inhibitor; hence α is replaced by the slowed rate of synthesis $\alpha_{i}$


$$\frac{dC}{dt}=\frac{\alpha_{i}}{v}-\beta C\left(t\right)$$

Dividing the equation by C_0_ and denoting $C^{\prime }=\frac{C}{C_{0}}$ :


$$\frac{dC^{\prime }}{dt}=\frac{\alpha_{i}}{vC_{0}}-\beta C^{\prime }\left(t\right)$$

At the new steady state ( t→∞) $\frac{dC^{\prime }}{dt}=0$ thus:


$$\frac{\alpha_{i}}{vC_{0}}=\beta C^{\prime }\left(\infty \right)$$

At the new steady state, $C^{\prime }\left(\infty \right)=\frac{\alpha_{i}}{\alpha }$ .

We denote k = $\frac{\alpha_{i}}{\alpha }$ such that *k* is the fractional protein synthesis rate in the presence of the inhibitor (for instance, *k* = 0.1 is equivalent to 10% of the nominal protein synthesis rate or 90% protein synthesis inhibition). Thus:


$$\frac{\alpha_{i}}{vC_{0}}=\beta k$$

$$\frac{dC^{\prime }}{dt}=\beta k-\beta C^{\prime }\left(t\right)$$

Solving the equation with the initial condition $C^{\prime }\left(0\right)=1$ results in:


$$C^{\prime }\left(t\right)=e^{-\beta t+\ln\left(1-k\right)}+k$$

H/M ratio reflects the residual amount of a particular protein pre-labeled with heavy AAs (H) after some time *t* in the presence of an inhibitor, divided by the residual amount of the same protein pre-labeled with medium AAs (M) in untreated neurons and the same *t*. Note that M should not be time-dependent as protein concentration is assumed to be at steady state (Step I above). Consequently, the expected H/M after some time *t* (in days) is given by:


$$\frac{H}{M}=e^{-\beta t+\ln\left(1-k\right)}+k$$

And


$$\log_{2}\left(\frac{H}{M}\right)=\log_{2}\left(e^{-\beta t+\ln\left(1-k\right)}+k\right)$$

Thus, given the half-life of a protein and the fold-change reduction of protein synthesis in the presence of a PSI, one can calculate the expected $\log_{2}\left(\frac{H}{M}\right)$ value.

Not surprisingly, correlations (r, Pearson’s) between expected and measured $\log_{2}\left(\frac{H}{M}\right)\;$ values improved with the duration of PSI exposure, as the predominance of measurement noise decreased. For the same reason, correlations were better for proteins with relatively short half-lives in which detectable changes might be expected following short PSIs exposure durations: For proteins with t_½_ ≤ 2 days: r = 0.177 (2 h); r = 0.341 (4 h); r = 0.463 (8 h). For proteins with t_½_ ≤ 10 days: r = 0.114 (2 h); r = 0.166 (4 h); r = 0.313 (8 h). All expected $\log_{2}\left(\frac{H}{M}\right)$ values are provided along measured ones in Supplementary Table S3.

### Annotation of protein categories and functions

Protein annotation was performed in Perseus (v1.5.1.6; Tyanova et al., 2016). The annotation was based on the annotation file: mainAnnot.rattus_norvegicus.txt.gz file (downloaded Apr. 10, 2022).^2^ Annotations were performed using the proteins’ Uniprot ID. The annotations were based on GOBP, GOCC, GOMF, and KEGG pathways. The selected search keywords for each of the functional groups listed in the Main Text are detailed in Supplementary Table S2B. The automatic software annotation was verified manually and refined where necessary (i.e., proteins for which automatic annotations were not properly backed by literature were excluded, and several proteins were added manually to the lists based on documented function / categories from current literature). These annotations were used to place volcano plot “hits” in specific categories / groups.

We also performed a GO enrichment analysis using GOrilla (Gene Ontology enRIchment anaLysis and visuaLizAtion tool; Eden et al., 2009).^3^ For this purpose, lists of all proteins conforming with the minimum requirement for biological repeats and minimum peptide numbers (the “stringent lists” described in Main Text) at each time point (0, 2, 4, and 8 h) were sorted separately by decreasing log_2_H/M ratios, with the most negative ratio at the top. Each list was subjected to GO based enrichment analysis according to GO Cellular Component, Molecular Function, and Biological Process with “Rattus Norvegicus” and default running parameters (single ranked gene list). GO terms which appeared at consecutive time points but were not enriched at the control (*t* = 0) were selected and plotted as a function of duration of PSI exposure. The sum of fold enrichments was calculated for each GO term and the top 25% enriched terms were selected (this was equivalent to sum fold enrichment of >12.7). The list was then filtered to exclude GO terms enriched only at one of the three time points (2, 4, 8 hours). Finally, we used QuickGO ^4^ to filter out high-level and /or redundant GO terms. For this purpose, we built an ancestor chart for the enriched terms and excluded the following from the visualization: (i) terms that were located higher than level 5 in the term hierarchies; and (ii) parent terms in those cases where both the child and parent terms were enriched.

Annotation of synaptic protein groups was done using an expert-curated knowledgebase for the synapse (SynGO; Koopmans et al., 2019). Bulk annotations were downloaded from the SynGO portal (bulk download SynGO release “20210225” annotations file, last modified May 17, 2022, downloaded Jun. 17, 2022).^5^ The data were merged based on first Human ortholog gene symbol. Annotations of the following groups were selected: Adhesion Molecules, Regulation of Synapse Organization, Active Zone, Postsynaptic Density, Presynapse, Synaptic Vesicle, and Postsynaptic Actin Cytoskeleton (GO terms included in each group are detailed in the legend of Figure 11B).

## Data availability statement

The mass spectrometry proteomics data have been deposited to the ProteomeXchange Consortium via the PRIDE partner repository (Perez-Riverol et al., 2022) with the dataset identifier PXD037859.

## Author contributions

LC and NEZ conceived and designed the study and wrote the first draft of the manuscript. LC performed the experiments and collected the data. LC and TZ performed the analysis and organized the databases. TZ wrote sections of the manuscript. All authors contributed to the article and approved the submitted version.

## Funding

This work was supported by funding to NEZ from the Israel Science Foundation (1470/18), the Deutsche Forschungsgemeinschaft (DFG) as part of funding of research unit ‘Syntophagy’ (5228), The Rappaport Institute and the Allen and Jewel Prince Center for Neurodegenerative Disorders of the Brain. LC was supported by a fellowship from the Gutwirth foundation.

## Conflict of interest

The authors declare that the research was conducted in the absence of any commercial or financial relationships that could be construed as a potential conflict of interest.

## Publisher’s note

All claims expressed in this article are solely those of the authors and do not necessarily represent those of their affiliated organizations, or those of the publisher, the editors and the reviewers. Any product that may be evaluated in this article, or claim that may be made by its manufacturer, is not guaranteed or endorsed by the publisher.

## Footnotes

^1^ http://cbl-gorilla.cs.technion.ac.il/

^2^ https://datashare.biochem.mpg.de/s/qe1IqcKbz2j2Ruf?path=%2FPerseusAnnotation%2FOrganismSpecific%2Fr

^3^ http://cbl-gorilla.cs.technion.ac.il/

^4^ https://www.ebi.ac.uk/QuickGO/annotations

^5^ https://syngoportal.org/
