# Supplementary material for: Synapse integrity and function: Dependence on protein synthesis and identification of potential failure points
Source: Front Mol Neurosci. 2022 Dec 13;15:1038614. doi: 10.3389/fnmol.2022.1038614 (PMC9792512; doi:10.3389/fnmol.2022.1038614)
Supplement: Supplementary file 6 [file Data_Sheet_1.pdf]

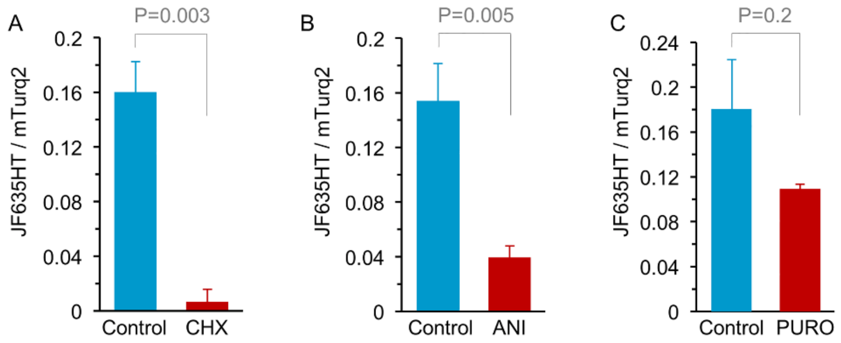

Supplementary Figure S1: Quantitative assessment of protein synthesis suppression following a 24-hour treatment with three protein synthesis inhibitors – analyses by replicate. Same data as Fig. 1, statistics pooled by replicate, rather than by neuron. **A)** 24-hour protein synthesis suppression by CHX. 4 replicates per condition from 2 separate experiments. **B)** 24-hour protein synthesis suppression by ANI. Data from 6 (ANI) and 7 (control, water) replicates from 3 separate experiments. **C)** 24-hour protein synthesis suppression by Puro. Data from 6 (Puro) and 4 (control, Puro. Aminonucleoside) replicates per condition from 3 and 2 separate experiments respectively. Scale bar, 20μm. Average + SEM, t-test assuming unequal variances. SEM and t test based on numbers of replicates.

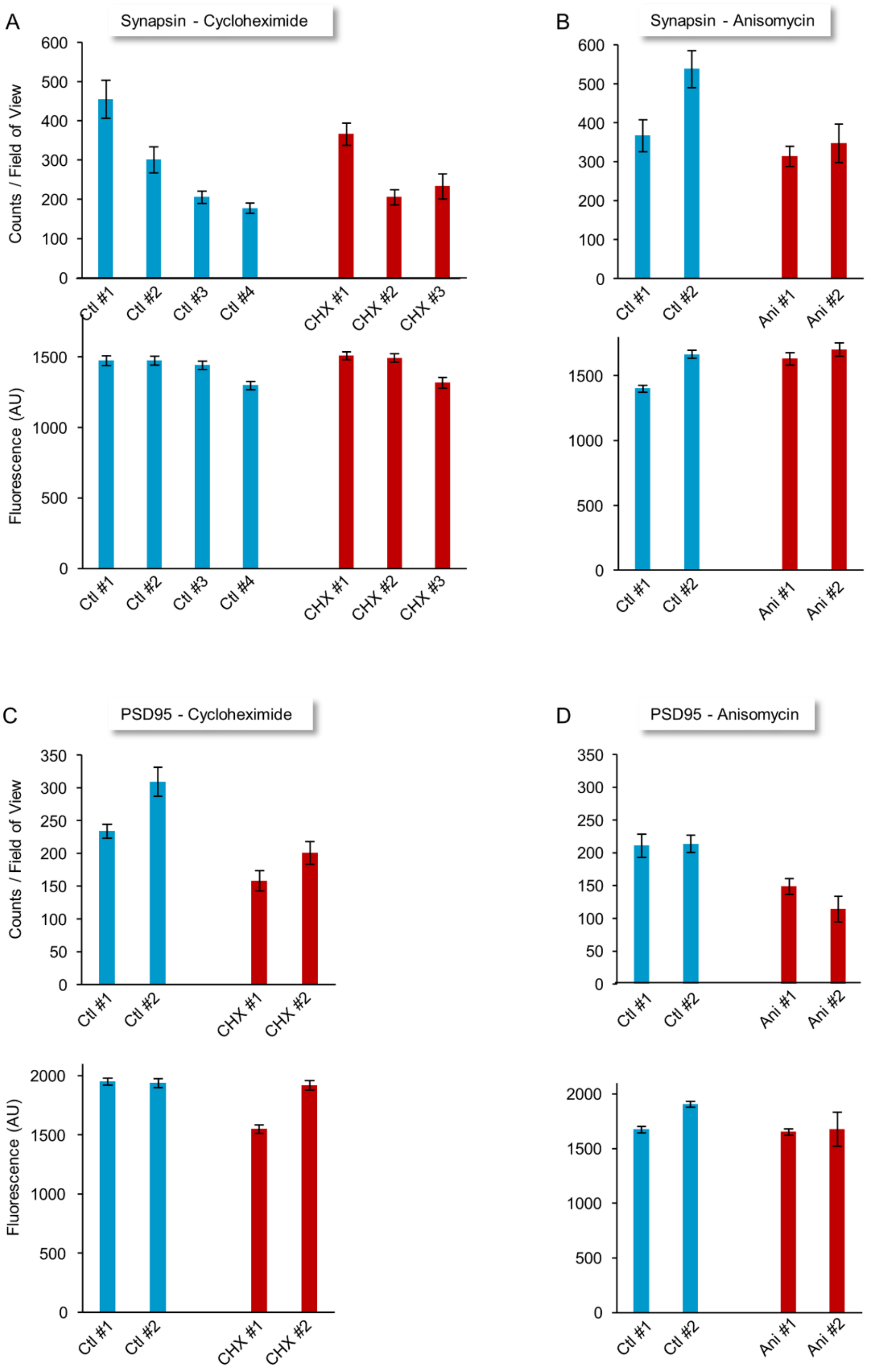

Supplementary Figure S2: Effects of protein synthesis inhibitor treatment on synaptic persistence - analyses by replicate. Cortical neurons grown for 2.5 weeks in 8mm diameter cylinders on coverslips were treated with protein synthesis inhibitors or vehicle solutions (controls) for 24 hours, fixed and stained with antibodies against the presynaptic protein Synapsin (**A**) or the postsynaptic protein PSD-95 (**B**). Puncta counts and mean fluorescence in each field view pooled for each coverslip (replicate).

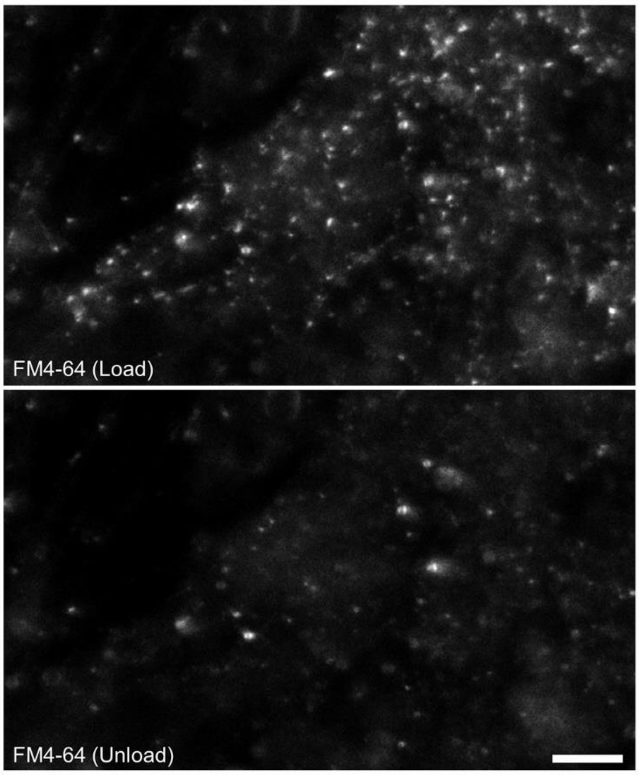

Supplementary Figure S3: Functional presynaptic sites are still present after 72 hours in the presence of PSIs. Neurons grown for 72 hours in the presence of CHX were labeled with FM4-64 by field stimulation (top) followed by dye unloading by field stimulation at 20 Hz for 120 sec. Note the presence of numerous FM-4-64 positive puncta in the top panel that lost most of their fluorescence upon unloading. Bar, 10 µm.

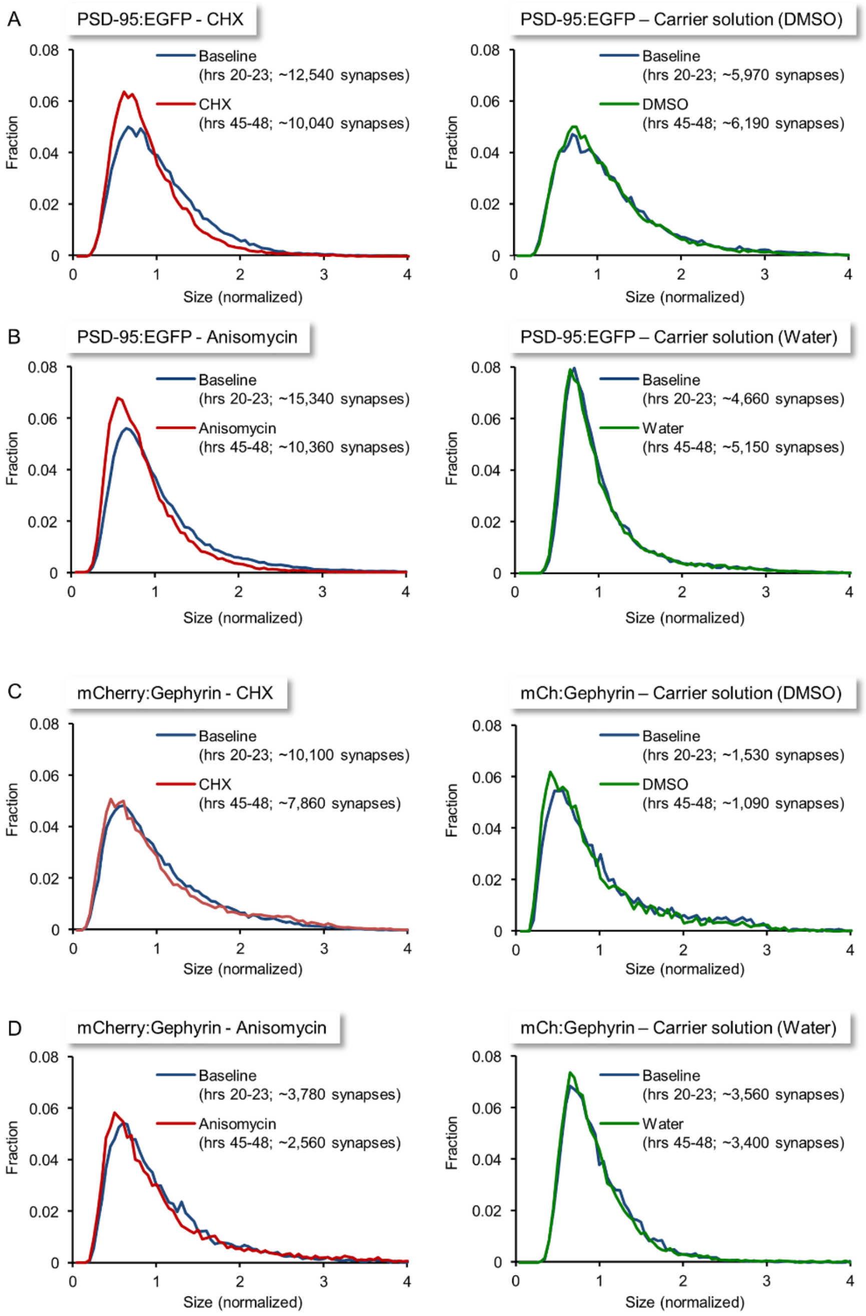

Supplementary Figure S4: Distributions of synaptic sizes before and after 24-hour PSI exposure. Normalized distributions of fluorescence intensities of PSD-95:EGFP and mCherry:Gephyrin puncta before (blue), and 24 hours after exposure to PSI (CHX/ANI; red) or carrier solutions (DMSO/water; green). Synaptic sizes were normalized, for each neuron, to the mean size of all synapses of that neuron, at t=0. **A,B)** Distributions of PSD-95:EGFP puncta fluorescence. CHX and carrier solution (**A**), ANI and carrier solution (**B**). **C,D)** Distributions of mCherry:Gephyrin puncta fluorescence. CHX and carrier solution (**C**), ANI and carrier solution (**D**). Total synapse numbers for each condition and time window are indicated alongside each panel.

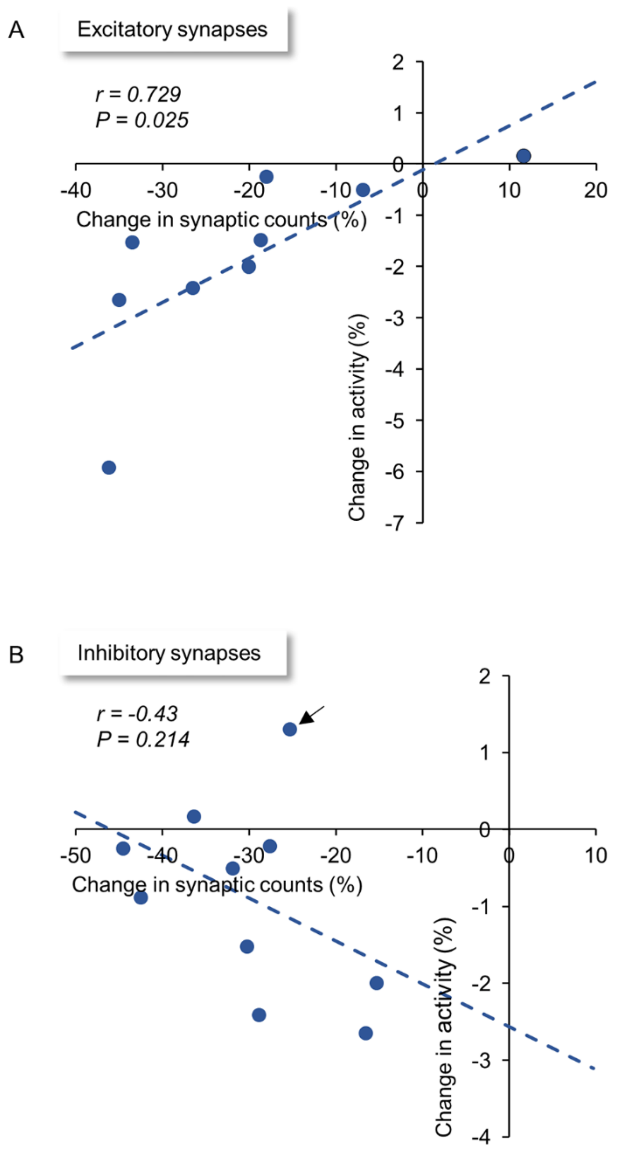

Supplementary Figure S5: Correlations between changes in excitatory or inhibitory synapse numbers and changes in spontaneous activity levels 24 hours post treatment. The % change in network activity (spontaneous spike rates measured from all MEA electrodes) 24 hours post treatment (as in Fig. 5D) was plotted against the extent of excitatory or inhibitory synapse loss (%) in the same networks over the same time window. **A)** Correlation between excitatory synapse loss and change in activity levels (r = 0.72, p = 0.02, n = 9 networks). **B)** Correlation between inhibitory synapse loss and change in activity levels. (r = -0.43, p = 0.21, n = 10 networks). Removing one outlier (arrow) resulted in a correlation of r = -0.69 (p = 0.04, n = 9 networks).

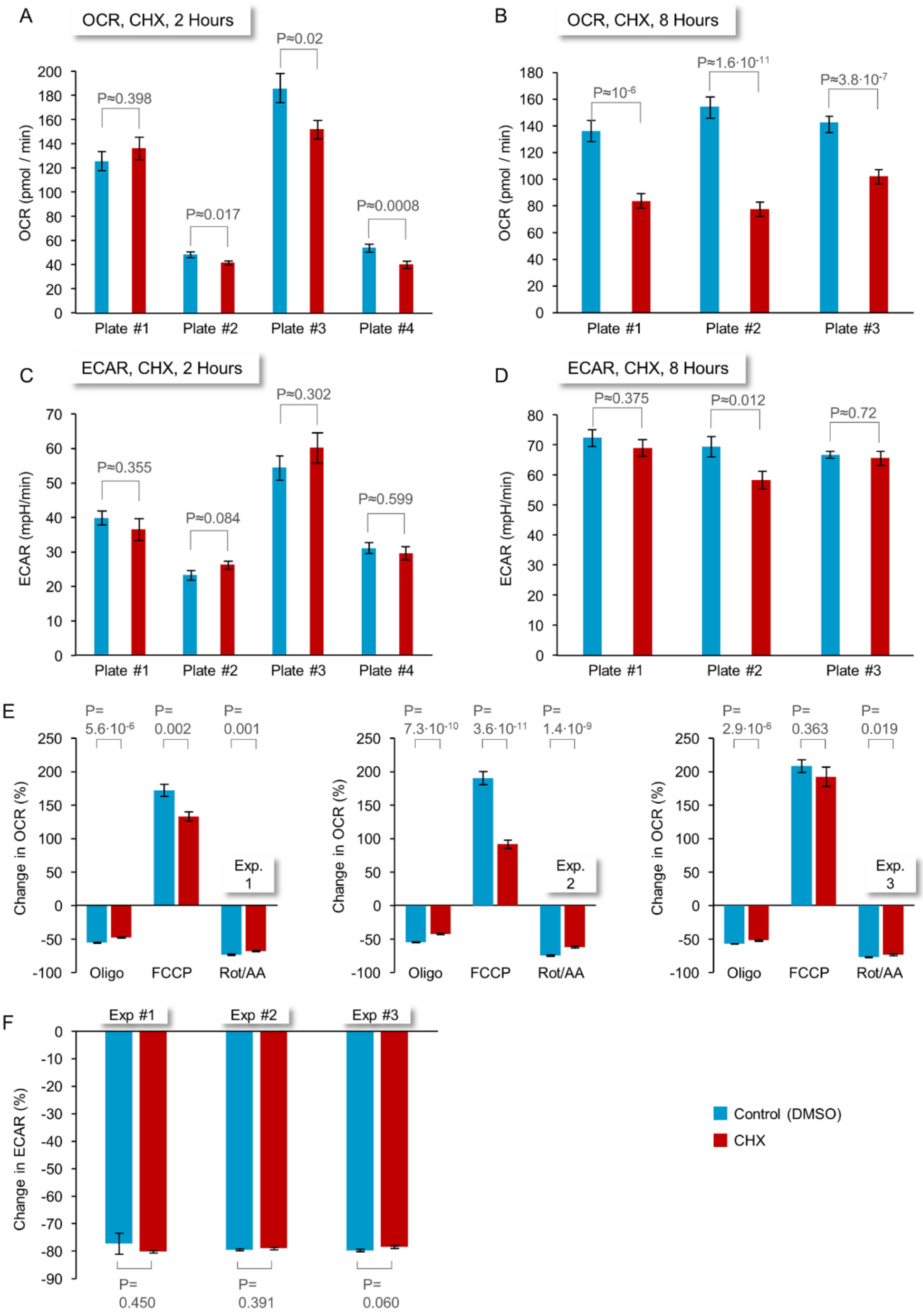

Supplementary Figure S6: Effects of PSI exposure on energy production capacity – data of separate experiments. Rat cortical neurons were plated on application-specific 96-well microplates. 18-21 days after plating, the neurons were treated with CHX or carrier solution (DMSO), for 2 or 8 hours. Oxygen consumption rates (OCR) and extracellular acidification rates (ECAR), measures of mitochondrial respiration and glycolysis, respectively, were then monitored in a Seahorse flux analyzer. **A)** Baseline OCR values. Preparations were treated for 2 hours with CHX or carrier solution (DMSO). 120 wells for each condition, 4 experiments, shown separately (each plate is from a separate experiment using independent cell culture preparations). **B)** Baseline OCR values. Preparations were treated for 8 hours with CHX or carrier solution (DMSO). 90 wells for each condition from 3 separate experiments. **C)** Baseline ECAR, 2 hours CHX or carrier solution. **D)** Baseline ECAR, 8

hours CHX or carrier solution. **E)** Sequential pharmacological manipulations reveal impairments in mitochondrial function after 8-hour protein synthesis suppression by CHX treatment. Cells were subjected sequentially to {oligomycin, carbonyl cyanide 4-(trifluoromethoxy) phenylhydrazone (FCCP), rotenone/antimycin A (Rot/AA) and 2-deoxyglucose (2-DG); see Materials and Methods for details}. Percent change in OCR, normalized to baseline level (3 experiments, 8 hours of exposure to CHX or carrier solution). **F)** Percent change in ECAR, normalized to baseline level. p-values are from two-sided t-tests assuming unequal variances. Error bars indicate ±SEM of microwells.

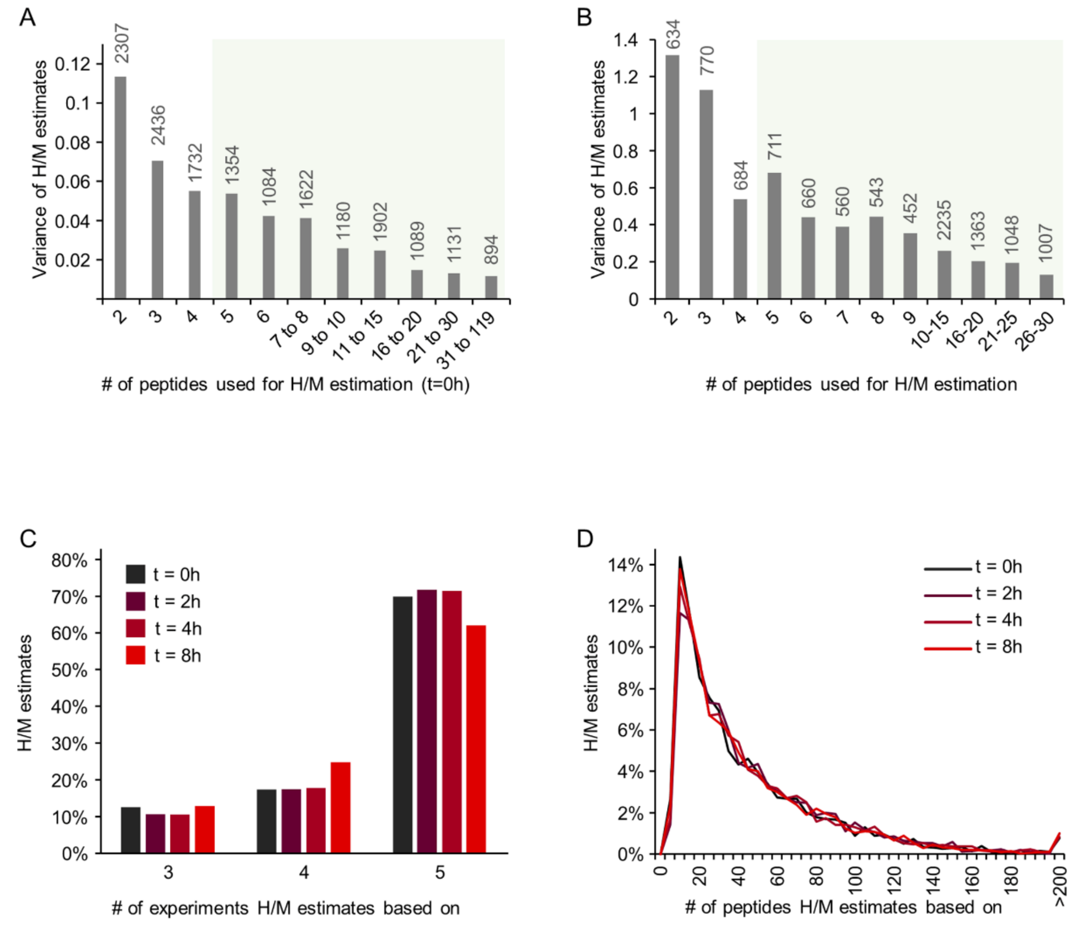

Supplementary Figure S7: Analysis of H/M estimate variability as functions of the peptide pair numbers identified by mass spectrometry. A) Variance of the $\log_2$H/M estimates as a function of number of peptides on which estimates are based. All data are from the control experiments (t=0). Number of reads in each bin is indicated above each bar. **B)** Mean variance of the $\log_2$H/M estimates for different ranges of peptide counts identified by the MS scan. Values were pooled from all time points and experiments (0,2,4,8 hours). Peptide counts were truncated at 30 for easier visualization (the full range was up to >400 peptides but the frequency of extremely abundant proteins was quite low). Number of reads in each bin is indicated above each bar. **C)** Number of experiments on which H/M estimates were based. **D)** Number of peptide pairs (per protein, per time point) on which H/M estimates were based. Data in panels **C,D** are from: 3856, 3629, 3633, 3552 proteins (time t=0, 2, 4, and 8 hours, respectively).

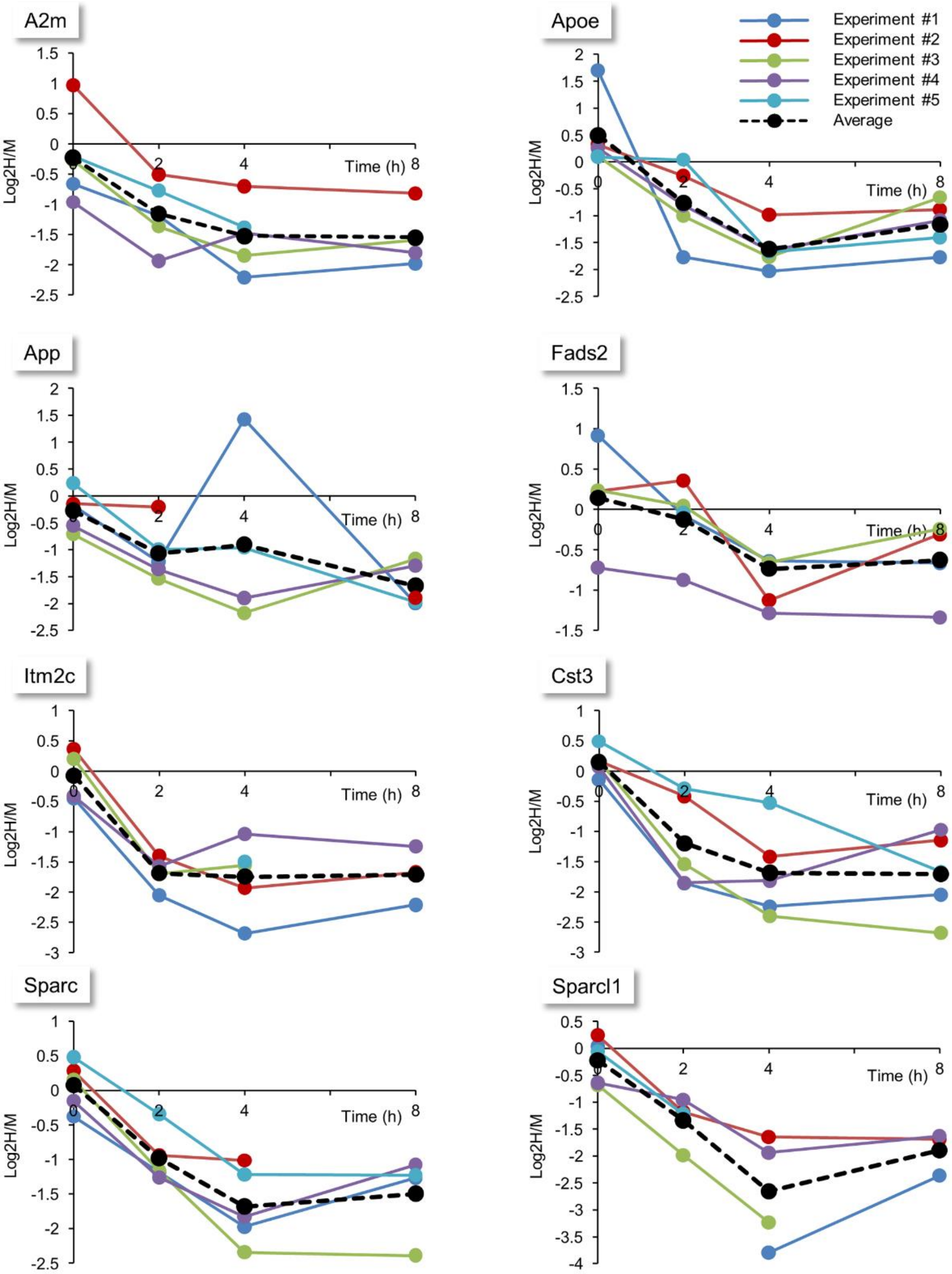

Supplementary Figure S8: Detailed time courses of protein loss. Log_2H/M values as a function of time are shown for eight proteins highlighted in Fig. 9B ("volcano plots"): A2m, Apoe, App, Fads2, Itm2c, Cst3, Sparc, Sparcl1. Each of the five biological repeats is shown in a different color. Black dashed lines indicate the mean values from all five experiments.

Supplementary Table Legends

Supplementary Table S1

Using SILAC and mass spectrometry to identify potentially essential proteins lost rapidly following protein synthesis suppression. The Log_2 fold-change at 2, 4, and 8 hours compared to t=0 and the statistical significance of the change are detailed for each protein. 'Biologically significant' fold-changes were considered as such if they were greater than two standard deviations of population Log_2 ratios at t=0 (~30% change) whereas statistically significant changes were considered as such if they passed a t-test for the biological repeats with a p value ≤ 0.05 (two-tailed unpaired t-test assuming equal variance). Two hours vs. 0 hours, 3629 proteins; 4 hours vs. 0h, 3633 proteins; 8 hours vs. 0h, 3552 proteins. Only proteins conforming to the criteria detailed in the Main Text (minimum peptide counts and biological repeats) at the control (t=0) and the relevant time point are included in these lists. Same data as in Fig. 9B.

Supplementary Table S2

A) Proteins exhibiting biologically significant changes following PSI exposure, with roles in key cellular functions and pathways. Among the proteins with significant changes: regulators of synapse stability / organization; proteins with roles in fatty acid / lipid metabolism; proteins related to cell bioenergetics; and proteins implicated in Alzheimer's disease (AD) pathology or colocalize with Aβ. Several of these proteins decreased consistently with increased exposure to the PSI. For each protein, the fold change at the relevant times following CHX exposure is indicated (values always indicate fold decrease, with the exception of Ndufs7, marked" UP" which increased following CHX exposure). Proteins marked by "#" were marginally significant at the denoted time points (i.e., borderline fold change, or borderline p-value). **B)** Search keywords for functional groups. For each of the four functions / categories: Synapse stability / organization; fatty acid / lipid metabolism; Cell bioenergetics; Alzheimer's disease (AD) pathology or colocalize with Aβ, the search keywords selected for a protein to be included in that group are indicated. Keywords were searched in the GOBP, GOCC, GOMF, and KEGG pathway annotations columns (for further details on the protein annotation, see Materials and Methods section).

Supplementary Table S3

Expected dependence of Log_2H/M ratios on protein half-life times. The measured Log_2H/M values and protein half-life times are indicated at all four time points (0, 2, 4, and 8 hours following PSI exposure; data on protein half-life times were obtained from Cohen *et al*, 2013; Hakim *et al*, 2016). Expected Log_2H/M values are indicated at 2, 4, and 8 hours following PSI exposure. Control (t=0 hours), 2549 proteins; t=2 hours, 2490 proteins; t=4 hours, 2502 proteins; t=8 hours, 2467 proteins. Data of Fig. 10B (only proteins with half-life times ≤ 10 days are shown in the Figure).

Supplementary Table S4

GO enrichment analysis of proteins whose levels were most strongly reduced following PSI exposure. A total of 341 GO terms enriched following 2, 4, or 8 hours of exposure to CHX. Enrichment analysis was done using GOrilla; GO terms already enriched at t=0 (control) were excluded (for further details, see Materials and Methods section). The GO terms are color coded as follows: GOCC – green; GOMF – blue; and GOBP – red. For each GO term at each time point (2, 4, and 8 hours), the Fold enrichment, p-value, FDR q-value, and number

of genes in the set, are indicated. The top 25% enriched terms, based on sum fold enrichment from all 3 time points, are shown in Fig. 11A.

Supplementary Table S5

Log_{10} rank (average of hours 2, 4, 8) of proteins with synaptic functions/categories following protein synthesis suppression. Synaptic function / category annotation was performed using SynGO (Koopmans *et al*, 2019) as detailed in the Main Text and Materials and Methods section. Data of Fig. 11B. Proteins in each synaptic protein group, and Log_{10} rank (average of hours 2, 4, 8 after PSI exposure), are indicated. Further details on the analysis, and relevant GO terms for each of the synaptic protein groups, are provided in the legend of Fig. 11B, and in the Materials and Methods section.
